# Evaluation of HIV treatment outcomes with reduced frequency of clinical encounters and antiretroviral treatment refills: A systematic review and meta-analysis

**DOI:** 10.1371/journal.pmed.1003959

**Published:** 2022-03-22

**Authors:** Noelle Le Tourneau, Ashley Germann, Ryan R. Thompson, Nathan Ford, Sheree Schwartz, Laura Beres, Aaloke Mody, Stefan Baral, Elvin H. Geng, Ingrid Eshun-Wilson

**Affiliations:** 1 Division of Infectious Diseases, School of Medicine, Washington University in St. Louis, Saint Louis, Missouri, United States of America; 2 Department of Epidemiology, Johns Hopkins Bloomberg School of Public Health, Baltimore, Maryland, United States of America; 3 Department of Global HIV, Hepatitis and Sexually Transmitted Diseases, World Health Organization, Geneva, Switzerland; 4 Centre for Infectious Disease Epidemiology and Research, School of Public Health and Family Medicine, University of Cape Town, Cape Town, South Africa; 5 Center for Dissemination and Implementation, Institute for Public Health, Washington University in St. Louis, Saint Louis, Missouri, United States of America; University of Southampton, UNITED KINGDOM

## Abstract

**Background:**

Global HIV treatment programs have sought to lengthen the interval between clinical encounters for people living with HIV (PLWH) who are established on antiretroviral treatment (ART) to reduce the burden of seeking care and to decongest health facilities. The overall effect of reduced visit frequency on HIV treatment outcomes is however unknown. We conducted a systematic review and meta-analysis to evaluate the effect of implementation strategies that reduce the frequency of clinical appointments and ART refills for PLWH established on ART.

**Methods and findings:**

We searched databases​ between 1 January 2010 and 9 November 2021 to identify randomized controlled trials (RCTs) and observational studies that compared reduced (6- to 12-monthly) clinical consultation or ART refill appointment frequency to 3- to 6-monthly appointments for patients established on ART. We assessed methodological quality and real-world relevance, and used Mantel–Haenszel methods to generate pooled risk ratios (RRs) with 95% confidence intervals for retention, viral suppression, and mortality. We evaluated heterogeneity quantitatively and qualitatively, and overall evidence certainty using GRADE. Searches yielded 3,955 records, resulting in 10 studies (6 RCTs, 3 observational studies, and 1 study contributing observational and RCT data) representing 15 intervention arms with 33,599 adults (≥16 years) in 8 sub-Saharan African countries. Reduced frequency clinical consultations occurred at health facilities, while reduced frequency ART refills were delivered through facility or community pharmacies and adherence groups. Studies were highly pragmatic, except for some study settings and resources used in RCTs. Among studies comparing reduced clinical consultation frequency (6- or 12-monthly) to 3-monthly consultations, there appeared to be no difference in retention (RR 1.01, 95% CI 0.97–1.04, *p =* 0.682, 8 studies, low certainty), and this finding was consistent across 6- and 12-monthly consultation intervals and delivery strategies. Viral suppression effect estimates were markedly influenced by under-ascertainment of viral load outcomes in intervention arms, resulting in inconclusive evidence. There was similarly insufficient evidence to draw conclusions on mortality (RR 1.12, 95% CI 0.75–1.66, *p =* 0.592, 6 studies, very low certainty). For ART refill frequency, there appeared to be little to no difference in retention (RR 1.01, 95% CI 0.98–1.06, *p =* 0.473, 4 RCTs, moderate certainty) or mortality (RR 1.45, 95% CI 0.63–3.35, *p =* 0.382, 4 RCTs, low certainty) between 6-monthly and 3-monthly visits. Similar to the analysis for clinical consultations, although viral suppression appeared to be better in 3-monthly arms, effect estimates were markedly influence by under-ascertainment of viral load outcomes in intervention arms, resulting in overall inclusive evidence. This systematic review was limited by the small number of studies available to compare 12- versus 6-monthly clinical consultations, insufficient data to compare implementation strategies, and lack of evidence for children, key populations, and low- and middle-income countries outside of sub-Saharan Africa.

**Conclusions:**

Based on this synthesis, extending clinical consultation intervals to 6 or 12 months and ART dispensing intervals to 6 months appears to result in similar retention to 3-month intervals, with less robust conclusions for viral suppression and mortality. Future research should ensure complete viral load outcome ascertainment, as well as explore mechanisms of effect, outcomes in other populations, and optimum delivery and monitoring strategies to ensure widespread applicability of reduced frequency visits across settings.

## Abstract 2

Noelle Le Tourneau and co-workers study HIV outcomes in studies investigating reduced frequency of antiretroviral drug provision and clinical consultations.

## Author summary

**Why was this study done?:**

- Global HIV services have moved toward differentiated service delivery (DSD) models that decrease the frequency of unneeded contact with the health system for patients established on antiretroviral treatment (ART) and ensure alignment between needs and services.
- It remains unclear, however, whether increasing ART clinical consultation intervals beyond 3 months, to 6 or 12 months, results in comparable treatment outcomes to 3-monthly visits.

**What did the researchers do and find?:**

- We conducted a systematic review and meta-analysis to identify studies that compared reduced (6- or 12-monthly) clinical consultation or ART refill appointment frequency to 3- or 6-monthly appointments for patients established on ART.
- We identified 10 studies representing 15 intervention arms with 33,599 adults in 8 sub-Saharan African countries, including 6 randomized controlled trials, 3 cohort studies, and 1 study contributing both observational and randomized data. The methodological quality of the randomized controlled trials was generally high, and cohort data were of high, fair, and poor quality.
- Among 8 studies comparing reduced clinical consultation frequency (6- or 12-monthly) to 3-monthly consultations, there appeared to be no difference in retention in care, and this result was consistent across study design and visit frequency. There was poor viral load outcome ascertainment in reduced frequency study arms; as a result, when including all randomized study participants, viral suppression appeared higher in 3-monthly compared to reduced frequency arms for RCTs, but when analyses were restricted to only patients with viral load measurement, there appeared to be no difference in viral suppression.
- Among studies comparing 6-monthly to 3-monthly ART refill frequency, there similarly appeared to be no difference in retention in care or mortality. Viral suppression also appeared higher in 3-monthly compared to reduced frequency refill arms but showed no difference when analysis was restricted to patients with viral load measurement.

**What do these findings mean?:**

- Extending clinical consultation intervals from 3 months to 6 or 12 months and ART dispensing intervals from 3 months to 6 months may result in similar outcomes for retention in care, with less robust conclusions for viral suppression and mortality.
- Future studies should aim to obtain more complete viral load outcome ascertainment in all study arms.
- To increase the applicability of these findings, further implementation research should explore other population groups, mechanisms of effect, and optimum delivery and monitoring strategies.

## Introduction

For people living with HIV (PLWH) in low- and middle-income countries (LMICs) and on antiretroviral treatment (ART), unnecessary clinic and pharmacy appointments impose an avoidable burden on both patients and providers. Efforts to decrease the frequency of unneeded contact with the health system represent a central pillar of the movement toward differentiated service delivery (DSD) models [1,2]. There is, however, concern that reduced frequency might also compromise meaningful clinical and psychosocial interactions with the health system and potentially, paradoxically, increase missed appointments or non-adherence over the long term. Most existing data support the hypothesis that longer intervals reduce obstacles to attending services for PLWH, including structural challenges such as the time and travel required to attend appointments and pick up medications—which result in considerable direct and indirect costs—as well as psychosocial barriers such as stigma [3–7].

Based on these early observations, and consistent with World Health Organization (WHO) recommendations issued in 2016, global HIV programs have moved away from monthly appointments (which were the norm for many years) to 3-monthly appointments. Questions have turned to whether extending appointment intervals beyond 3 months to 6 or 12 months may be safe and effective. Early data syntheses suggesting that outcomes are equivalent for 3- versus 1-monthly appointments [8,9] do not automatically apply to further extensions. Qualitative, survey, and preference data indicate that PLWH value DSD models that include lengthened appointment intervals [4,10,11], but also that some PLWH do prefer more frequent psychosocial support from interactions with their providers [3]. Implementation of reduced visit frequency has accelerated pace in the past 2 years as it has become a practical necessity during the COVID-19 pandemic to decongest health facilities and limit physical contact [12,13]. Understanding how visit frequency impacts HIV treatment outcomes and under what conditions differences in outcomes manifest could further inform the implementation of this DSD strategy.

To support the 2021 update of the WHO service delivery guidelines [14], we undertook a systematic review and meta-analysis, including observational studies and randomized controlled trials (RCTs), in which we explicitly examined outcomes for clinical and ART refill visit intervals of 6 months or greater compared to 3 months. We present the data that contributed to the WHO guideline update as well as recently published studies. Such syntheses regarding further reductions in appointment frequency not only inform normative guidance on evolving DSD approaches but have particular salience for healthcare in the era of a pandemic.

## Methods

The protocol is registered in PROSPERO (CRD42019128609).

### Eligibility criteria

We included individual and cluster RCTs, comparative observational studies, cross-sectional studies, and single arm intervention studies without a comparison group. We included studies that enrolled PLWH established on first-line ART in LMICs. The definition of being established on ART varied by study (see Results). We included studies that reported outcomes of retention in care, viral suppression, and mortality. Eligible interventions included those with any component of less frequent clinical consultation or less frequent ART dispensing visits (e.g., 6- or 12-monthly) compared to 3- or 6-month frequency intervals (Table 1). Studies reporting 2-month comparison intervals were classified as 3-monthly for the purposes of this review.

**Table 1:** Eligibility criteria for included studies.

PICO criterion	Description
Population	People living with HIV established on first-line ART as defined by study, in low- and middle-income countries
Intervention	Less frequent clinical or ART dispensing appointments (e.g., 6 or 12 months)
Comparison	3- or 6-monthly clinical or ART dispensing appointments
Outcome	Retention in care as defined by study; viral suppression as defined by study; mortality

ART, antiretroviral treatment; PICO, population, intervention, comparison, and outcome.

### Search strategy and selection criteria

We searched MEDLINE (PubMed), Embase (OVID), Cochrane Central Register of Controlled Trials, WHO International Clinical Trials Registry Platform (ICTRP), and ClinicalTrials.gov from 1 January 2010 through 9 November 2021, as well as Conference on Retroviruses and Opportunistic Infections (CROI) from 2017 to 2021 and International AIDS Society (IAS) conferences from 2016 to 2021. We additionally reviewed references, consulted experts in the field, and reviewed the IAS DSD resources [15].

### Data extraction and quality assessment

Abstracts and titles were screened by 2 authors (NL, AG, RRT) in duplicate in Covidence [16], with any discrepancies resolved by a third author (IEW). Data on the study setting and population, intervention, and outcomes of eligible studies were extracted into an online database platform Airtable (https://airtable.com), with quality assurance of data done by a second author (NL or AG). Outcomes were extracted with numerators and denominators, as well as measures of association when possible. Reporting of study outcome quality was also extracted in Airtable. We assessed risk of bias using the Cochrane risk of bias tool (RoB-1) for RCTs and additionally judged risk of bias for each outcome as “low risk,” “high risk,” or “some concerns” [17,18]. We applied the Newcastle–Ottawa Scale to observational studies, with studies categorized with regard to risk of bias as “good quality,” “poor quality,” or “fair quality” [19]. We evaluated heterogeneity qualitatively and quantitatively through stratified analysis, and used GRADE to evaluate overall evidence certainty. We used PRECIS-2 criteria to assess how pragmatic or explanatory included studies were.

### Intervention categorization

Increased spacing of clinical assessments and ART refills was frequently a component of broader DSD interventions. We therefore characterized interventions according to the frequency of clinical assessment, the location of clinical assessment, the health worker providing the clinical assessment, the frequency of ART refills, the location of refills, the delivery method of refills, and who was providing refills. We conducted separate analyses to evaluate outcomes associated with (1) reduced clinical appointment frequency and (2) reduced ART refill dispensing frequency.

### Outcome definition

The primary outcome was retention in care, defined as the proportion of individuals retained on ART and in care at last available follow-up. Secondary outcomes were documented viral suppression at last available follow-up, and mortality. The viral load threshold for defining viral suppression was determined by the authors of each study reporting viral suppression. Planned secondary outcomes of adherence and morbidity were not assessed due to limited reporting in included studies.

### Statistical analysis

We conducted pairwise meta-analysis comparing (1) reduced clinical assessment frequency with either 3-monthly or 6-monthly clinical assessments and (2) reduced ART refill dispensing frequency with 3-monthly ART refill frequency. For studies with more than 1 treatment arm, we split the comparison arm if both treatment arms were included in the pooled estimate. For analysis, we included numerators and denominators reported from individual studies and cluster-adjusted estimates ​for cluster RCTs based on intraclass correlation coefficients (ICCs) from the literature [20–23], according to methodology outlined in Cochrane guidelines [17], to generate overall risk ratios (RRs) with 95% confidence intervals (CIs) by study design. When adjusted measures of associations were available, we pooled relative data for studies reporting time-to-event data as hazard ratios (HRs). Data were synthesized using R programming (packages “metafor” and “metabin”) using Mantel–Haenszel methods for pooling and random effects. Subgroup analyses with pooled RRs were performed where appropriate, including for different frequencies of refills or clinical consultations and different delivery strategies.

## Results

### Search and screening results

**Fig 1:**
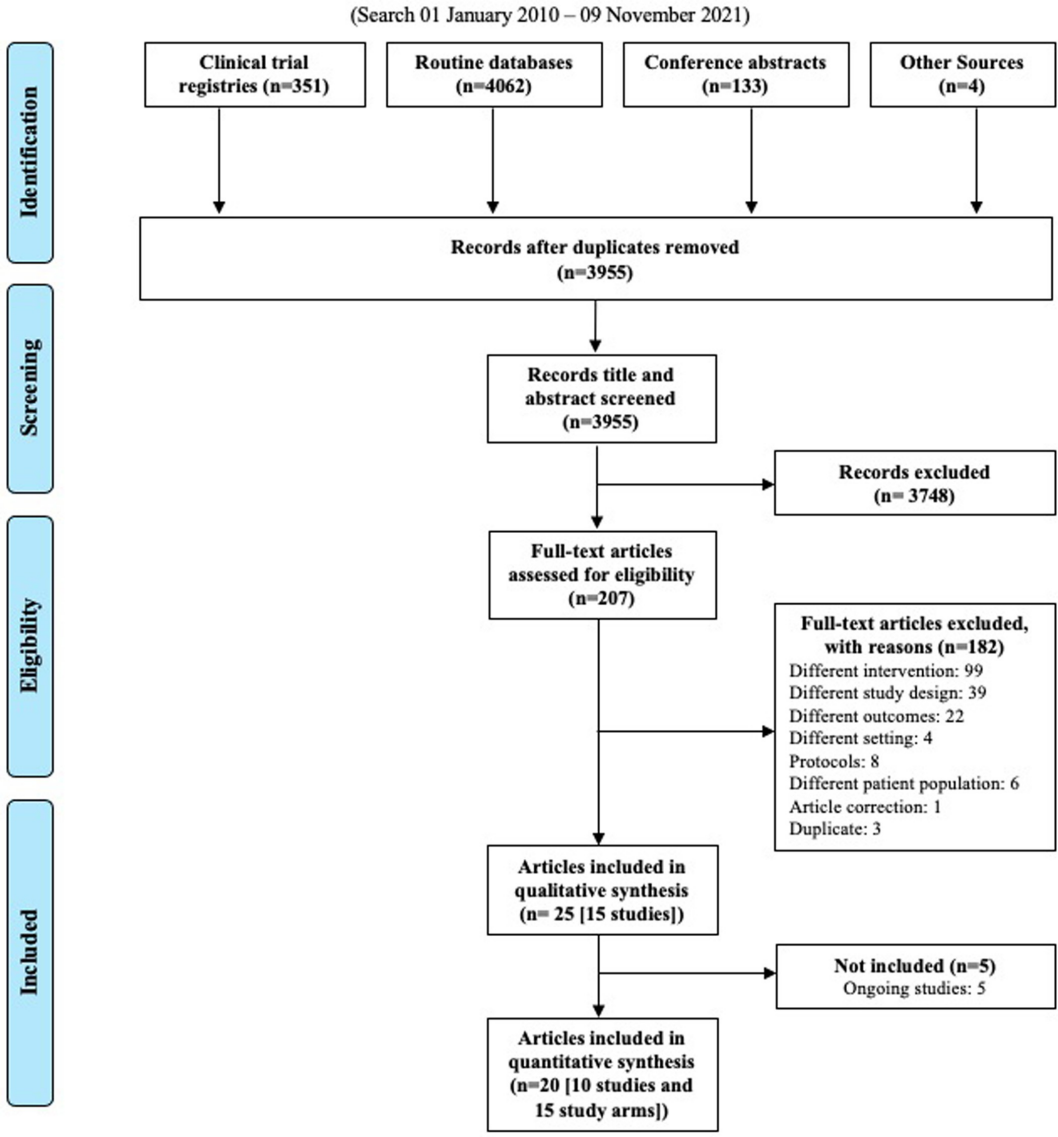
PRISMA search results.

Searches yielded 3,955 records after deduplication; these 3,955 records underwent title and abstract screening. In total, 207 full-text articles were assessed for eligibility, and 20 records representing 10 studies with 15 intervention study arms met the criteria for inclusion in our review (Fig 1).

### Included studies

The 10 eligible studies including 15 intervention arms included 6 RCTs, 3 comparative observational studies, and 1 study that contributed both observational and randomized data. All studies were conducted in sub-Saharan Africa, with a total of 33,599 participants across all studies. Two studies included PLWH aged ≥16 years, the rest included PLWH aged ≥18 years. We detected substantial clinical heterogeneity, including variability in the intervention components, the contexts in which reduced visit frequency was delivered, and the interval lengths between clinical consultations and ART refills. Eight intervention study arms with 6-monthly clinical consultations and 7 intervention study arms with 12-monthly clinical consultations were included. Four studies had intervention study arms with 6-monthly ART dispensing intervals. Characterization of the intervention strategies to reduce facility contact included 3 studies with ART dispensation at the clinic, 6 studies with ART dispensation in the community, and 4 studies with ART dispensation in the community or clinic. The majority of the studies (*n* = 8) included an “adherence club” component as part of the reduced appointment frequency strategy, where PLWH met in either a community or clinic setting. Other interventions included ART dispensation in private pharmacies and venues in the community and ART dispensation at home visits. Individual study and intervention details are summarized in Tables 2 and 3. Ten studies contributed to meta-analyses of reduced clinical consultation frequency (with 15 intervention study arms in total), and 4 studies contributed to meta-analyses of reduced ART refill frequency (4 intervention study arms). There was substantial methodological heterogeneity with regard to study design, risk of bias, and outcome assessment measures.

**Table 2:** Summary of included studies.

Study	Design	Country	Setting	Participants	*N*	“Established on ART” population definition and inclusion criteria
Cassidy 2020 [24–26]	Randomized (cluster, parallel)	South Africa	Peri-urban	≥18 years	2,150	ART > 6 months, VL < 400, no OI, non-pregnant; already in an existing adherence club
Fatti 2020a [22,27–29]	Randomized (cluster, parallel)	Zimbabwe	Urban, rural	≥18 years	2,295	ART ≥6 months, VL ≤ 1,000; weight ≥ 35 kg
Fatti 2020b [22,27–29]	Randomized (cluster, parallel)	Zimbabwe	Urban, rural	≥18 years	2,505	ART ≥6 months, VL ≤ 1,000; weight ≥ 35 kg
Fox 2019a [20]	Randomized (cluster, parallel)	South Africa	Urban, rural	≥18 years	569	ART ≥12 months, VL in past 3 months, 2 undetectable VL measurements (<400), non-pregnant
Fox 2019b [20]	Prospective cohort	South Africa	Urban, rural	≥18 years	578	ART ≥12 months, VL in past 3 months, 2 undetectable VL measurements (<400), non-pregnant
Goodrich 2021 [30,31]	Randomized (cluster, parallel)	Kenya	ND	≥18 years	420	ART ≥6 months, CD4 > 200, VL < 40, no OI, non-pregnant
Grimsrud 2016 [32,33]	Prospective cohort	South Africa	Urban	≥16 years	8,150	ART >12 months, 2 suppressed VL measurements (<400), no OI
Hoffman 2021 [34,35]	Randomized (cluster, parallel)	Malawi, Zambia	Urban, rural	≥18 years	8,719	ART ≥6 months, VL < 1,000 in Malawi or VL < 20 in Zambia in past 6 months, no OI, non-pregnant
Nichols 2021a [36]	Retrospective cohort	Zambia	Urban, rural	≥18 years	1,146	WHO stage 0–2, no OI, 12 months of follow-up
Nichols 2021b [36]	Retrospective cohort	Zambia	Urban	≥18 years	585	WHO stage 0–2, no OI, 12 months of follow-up
Nichols 2021c [36]	Retrospective cohort	Zambia	Urban, rural	≥18 years	561	WHO stage 0–2, no OI, 12 months of follow-up
Pasipamire 2018 [37]	Retrospective cohort	Swaziland	Rural	≥16 years	918	ART ≥12 months, VL suppression
Tukei 2020a [28,29,38,39]	Randomized (cluster, parallel)	Lesotho	Urban, rural	≥18 years	2,507	ART ≥6 months, VL < 1,000 in past 12 months
Tukei 2020b [28,29,38,39]	Randomized (cluster, parallel)	Lesotho	Urban, rural	≥18 years	2,829	ART ≥6 months, VL < 1,000 in past 12 months
Woodd 2014 [40]	Randomized (cluster, parallel)	Uganda	Urban, rural	≥18 years	1,453	Not necessarily established on ART; ART eligibility: WHO HIV clinical stage 4 or late stage 3 or CD4 < 200

ND, not described; OI, opportunistic infection; VL, viral load; WHO, World Health Organization. CD4 cell count measure unit, cells/μL; VL measure unit, copies/mL.

**Table 3:** Summary of intervention characteristics.

Study*	Reduced frequency group								Facility-based comparator group(s)
	Clinical assessment appointment			ART dispensing visit				Extra support	
	Frequency	Location	Clinician	Frequency	Location	Method	Who delivers		
Cassidy 2020 (i)	12 mo	Clinic	ND	6 mo	Community or clinic	AC	ND	—	—
Cassidy 2020 (ii)	~6 mo	Clinic	ND	~2.5 mo (5/year)	Community or clinic	AC	ND	—	—
Fatti 2020a	12 mo	Clinic	ND	3 mo	Community	AC	AC member	—	3-mo facility ART collection and clinical consultation
Fatti 2020b	12 mo	Clinic	ND	6 mo	Community	AC	AC member	—	3-mo facility ART collection and clinical consultation
Fox 2019a	6 mo	Clinic	ND	2–3 mo	Community or clinic	AC	Lay staff, nurses	—	2-mo ART refill at clinic, counseling, support groups; 4 sites had AC as part of SOC
Fox 2019b	6 mo	Clinic	ND	ND	Community	Private pharm, venues	ND	—	2-mo ART refill at clinic, counseling, support groups; 4 sites had AC as part of SOC
Goodrich 2021	12 mo	Clinic	ND	3 mo	Community	AC	ND	Adherence support	3- to 4-mo clinic appointments
Grimsrud 2016	12 mo	Community	Nurse	~2.5 mo (5/year)	Community or clinic	AC	CHW	Group counseling	2-mo ART refill at clinic appointment
Hoffman 2021 (i)	6 mo	Clinic	Provider	6 mo	Clinic	Pharm	ND	—	—
Hoffman 2021 (ii)	3 mo	Clinic	Provider	3 mo	Clinic	Pharm	ND	—	—
Nichols 2021a	6 mo	Clinic	ND	1 mo	Community	AC	AC member	—	3-mo facility ART collection and clinical consultation
Nichols 2021b	6 mo	Clinic	ND	2–3 mo	Clinic	AC	Lay HCW	ART counseling	3-mo facility ART collection and clinical consultation
Nichols 2021c	6 mo	Clinic	ND	1–3 mo	Home	Home visits	CHW	Health screening, adherence support	3-mo facility ART collection and clinical consultation
Pasipamire 2018 (i)	6 mo	Clinic	ND	1 mo	Community	AC	AC member	ART counseling	—
Pasipamire 2018 (ii)	3 mo	Clinic	ND	3 mo	Clinic	AC	ND	Peer education sessions, ART adherence info	—
Pasipamire 2018 (iii)	ND	ND	ND	ND	Community	Mobile clinic outreaches	ND	Ante/post-natal health services	—
Tukei 2020a	12 mo	Clinic	ND	3 mo	Community	AC	AC member	—	3-mo facility ART collection and clinical consultation
Tukei 2020b	12 mo	Clinic	ND	6 mo	Community	Community outreach post	CHW	—	3-mo facility ART collection and clinical consultation
Woodd 2014	6 mo	Clinic	Clinician	1 mo	Home	Home visits	Lay workers	Peer support	1-mo ART refills at clinic, 3-mo clinician appointments, adherence support

AC, adherence club; ART, antiretroviral treatment; CHW, community health worker; CO, clinical officer; HCW, healthcare worker; mo, monthly; ND, not described; pharm, pharmacy; SOC, standard of care.

*For studies with active comparator arms that differ from a facility-based comparator group: (i) reduced frequency arm; (ii, iii) active comparator arms.

### Risk of bias

As assessed by risk of bias tools, data from RCTs were generally judged as having high methodological quality (low risk of bias) or some concerns, and data from the 3 observational studies were judged as having high quality, fair quality, and poor quality (Tables 4 and S3). Data from cohort studies were considered fair or poor quality primarily due to the comparison arm comprising a patient population with different eligibility than the intervention arm (e.g., different levels of “stability”) [32,37]. RCTs were judged as having some concerns when those enrolled did not meet eligibility criteria [25], those eligible for the intervention did not necessarily receive it [20], or there was high withdrawal in the intervention arm [31].

**Table 4:** Summary of risk of bias assessment.

Study	Retention in care	Viral suppression among randomized	Viral suppression among analyzed	Mortality
Cassidy 2020*	Some concerns	Some concerns	Some concerns	Some concerns
Fatti 2020a*	Low risk	Low risk	Low risk	Low risk
Fatti 2020b*	Low risk	High risk	Low risk	Low risk
Fox 2019a*	Some concerns	Some concerns	Some concerns	Not reported
Fox 2019b**	High quality	High quality	High quality	Not reported
Goodrich 2021*	Some concerns	High risk	Some concerns	High risk
Grimsrud 2016**	Fair quality	Fair quality	Fair quality	Not reported
Hoffman 2021*	Low risk	Not reported	Not reported	Low risk
Nichols 2021a**	High quality	Not reported	Not reported	Not reported
Nichols 2021b**	High quality	Not reported	Not reported	Not reported
Nichols 2021c**	High quality	Not reported	Not reported	Not reported
Pasipamire 2018**	Poor quality	Not reported	Not reported	Poor quality
Tukei 2020a*	Low risk	Low risk	Low risk	Low risk
Tukei 2020b*	Low risk	High risk	Low risk	Low risk
Woodd 2014*	Low risk	Not reported	Not reported	Low risk

*Assessments based on Cochrane risk of bias tool (RoB-1) for randomized controlled trials: high risk (red), some concerns (yellow), or low risk (green).

**Assessments based on Newcastle–Ottawa Scale for cohort studies: poor quality (red), fair quality (yellow), or high quality (green).

### PRECIS-2 score

Overall, studies were highly pragmatic as they were conducted in real-world settings with few additional measures to guarantee adherence to ART beyond what would occur in routine practice (Table 5). RCTs were on average less pragmatic than cohort studies. Studies were downgraded when they required extensive expertise or organization to deliver the intervention of reduced frequency clinical assessments or reduced frequency ART refills, such as in the case of home visits or group adherence clubs. Studies were less pragmatic when the intervention was delivered at clinical sites associated with research or at a single site.

**Table 5:** Summary of PRECIS-2 score.

Study	Study design	Eligibility	Recruitment	Setting	Organization	Flexibility: Delivery	Flexibility: Adherence	Follow-up	Primary outcome	Primary analysis
		Who is selected to participate in the trial?	How are participants recruited into the trial?	Where is the trial being done?	What expertise and resources are needed to deliver the intervention?	How should the intervention be delivered?	What measures are in place to make sure participants adhere to the intervention?	How closely are participants followed up?	How relevant is it to participants?	To what extent are all data included?
Cassidy 2020	RCT (cluster)	4	4	5	3	3	4	5	5	5
Fatti 2020	RCT (cluster)	4	4	3	4	4	5	5	4	5
Fox 2019	Cohort	4	4	5	5	4	5	5	5	4
Goodrich 2021	RCT (cluster)	5		4	3	4		5	5	5
Grimsrud 2016	Cohort	5	5	5	5	4	5	5	5	5
Hoffman 2021	RCT (cluster)	4	4	4	4	4	5	5	5	5
Nichols 2021	Cohort	4	5	4	3	4	5	5	5	5
Pasipamire 2018	Cohort	5		3	4			5	4	5
Tukei 2020	RCT (cluster)	4	5	3	4	4	5	5	5	5
Woodd 2014	RCT (cluster)	5	5	4	2	5	4	5	5	4

RCT, randomized controlled trial. A value of 5 (dark green) represents a very pragmatic approach, and a value of 1 (yellow) represents a very explanatory approach.

### Reduced clinical consultation frequency

#### Retention in care

**Fig 2:**
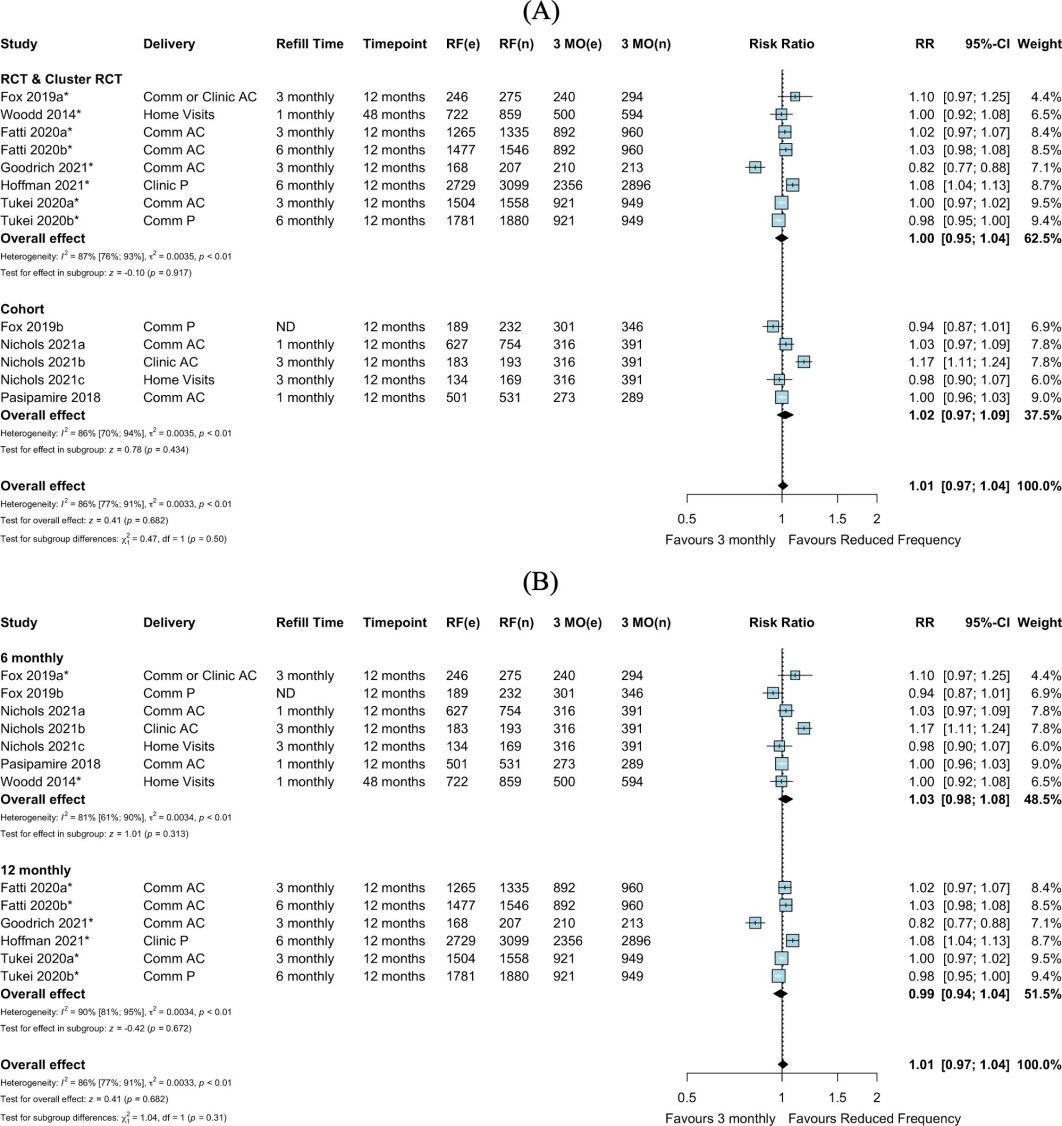
Retention in care: Reduced frequency versus 3-monthly clinical consultations among randomized or enrolled individuals. (A) By study design. (B) By clinical consultation frequency. Goodrich 2021 had high withdrawal from the intervention (community-based care) arm; it was unclear if those who withdrew reengaged in care at the health facility. Fox 2019a and Fox 2019b were separated into an RCT and cohort design, respectively, based on the analysis described by the authors, where randomization was not preserved in the intervention arm in Fox 2019b. *Cluster-adjusted RR. 3 MO, 3-monthly; AC, adherence club; Comm, community; e, number of events; n, number of participants; ND, not described; P, pharmacy; RCT, randomized controlled trial; RF, reduced frequency; RR, risk ratio.

We identified 9 comparative studies including 2 cohort studies, 6 randomized trials, and 1 study contributing both observational and RCT data (contributing 14 intervention arms in total) that reported retention in care and were included in the pairwise meta-analysis. Retention outcomes were reported by electronic health records and/or chart review, with the definition of retention in care provided by the authors (S1 Table). Among 8 studies with 13 total intervention arms comparing reduced clinic consultation frequency (>3-monthly) to 3-monthly clinical consultations, there appeared to be no difference in retention among all randomized individuals (RR 1.01, 95% CI 0.97–1.04, *p =* 0.682), consistent in both RCTs and observational data (RR 1.00, 95% CI 0.95–1.04, *p =* 0.917, and RR 1.02, 95% CI 0.97–1.09, *p =* 0.434, respectively) (Fig 2A). These findings were also consistent when stratified by 6- or 12-monthly clinical consultations (RR 1.03, 95% CI 0.98–1.08, *p =* 0.313, and RR 0.99, 95% CI 0.94–1.04, *p =* 0.672, respectively) (Fig 2B) and delivery strategy (S1 Fig). There was substantial statistical heterogeneity in study design and clinical consultation frequency subgroups, in part due to the inclusion of 1 study with high withdrawal from the intervention (community-based care) arm [31]. Among studies rated as high quality or having low risk of bias, heterogeneity remained substantial (S2 Fig). In exploration of the heterogeneity of study-specific definitions of established-on-ART patient populations, there remained substantial statistical heterogeneity across subgroups of required time spent on ART (12 months, 6 months, or other) for eligibility (S3 Fig).

**Fig 3:**
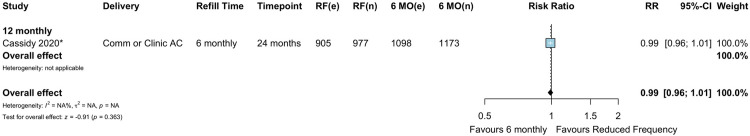
Retention in care: Reduced frequency versus 6-monthly clinical consultations. *Cluster-adjusted RR. 6 MO, 6-monthly; AC, adherence club; Comm, community; e, number of events; n, number of participants; RF, reduced frequency; RR, risk ratio.

Only 1 study compared 12-monthly clinical consultations to 6-monthly consultations [25]; this study reported similar retention in care at 24 months for 12- and 6-monthly clinical consultations (RR 0.99, 95% CI 0.96–1.01, *p =* 0.363) (Fig 3).

#### Viral suppression

**Fig 4:**
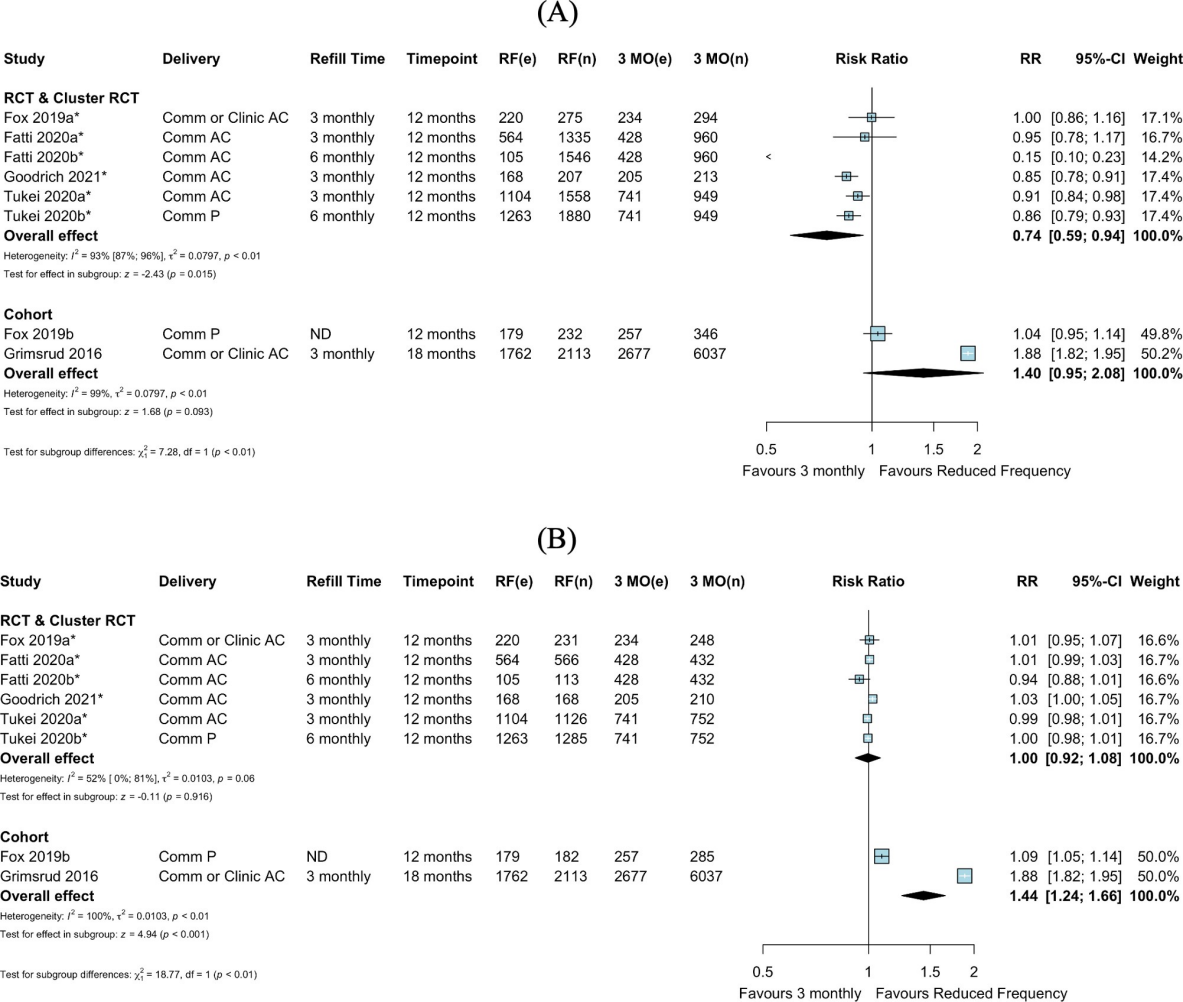
Viral suppression: Reduced frequency versus 3-monthly clinical consultations, by study design. (A) Among those randomized or enrolled. (B) Among those with viral load testing. Fox 2019a and Fox 2019b were separated into an RCT and cohort design, respectively, based on the analysis described by the authors, where randomization was not preserved in the intervention arm in Fox 2019b. *Cluster-adjusted RR. 3 MO, 3-monthly; AC, adherence club; Comm, community; e, number of events; n, number of participants; ND, not described; P, pharmacy; RCT, randomized controlled trial; RF, reduced frequency; RR, risk ratio.

We identified 6 comparative studies—1 cohort study, 4 randomized trials, and 1 study contributing both observational and RCT data (contributing 9 total arms for comparison)—that reported viral suppression for inclusion in the pairwise meta-analysis. Viral suppression outcomes were reported by electronic health records and/or chart review using variable thresholds (<400 copies/ml and <1,000 copies/ml) (S1 Table). RCT meta-analysis suggested decreased viral suppression for reduced frequency of clinical consultations compared to 3-monthly clinical consultations (RR 0.74, 95% CI 0.59–0.94, *p =* 0.015), while cohort studies showed slightly greater viral suppression for reduced frequency of clinical consultations compared to 3-monthly clinical consultations (RR 1.40, 95% CI 0.95–2.08, *p =* 0.093) (Fig 4A). Within RCT and cohort subgroup analyses, substantial statistical heterogeneity persisted (Fig 4A), markedly influenced by 1 RCT where only 7.3% of the reduced frequency arm received viral load testing and 1 cohort study with substantially higher viral suppression among those in the reduced frequency arm (there were substantial baseline imbalances between study arms in this study, with those receiving reduced visit frequency on ART for longer periods than those in the 3-monthly arm) [27,32] (S2 Table). As estimates differed by study design, overall estimates were not pooled across RCTs and cohort studies in subgroup analyses for clinical consultation frequency (6- or 12-monthly) and delivery strategy (S4 and S6 Figs).

In an available case analysis (including only those who received viral load testing), there appeared to be similar viral suppression in the arms for reduced frequency and 3-monthly clinical consultations among RCTs (RR 1.00, 95% CI 0.92–1.08, *p =* 0.916), and possible improved viral suppression for reduced frequency clinical consultations compared to 3-monthly clinical consultations among cohort studies (RR 1.44, 95% CI 1.24–1.66, *p* < 0.001) (Fig 4B). Due to differences in the pooled estimates in RCTs and cohorts, estimates were not pooled across study designs in subgroup analyses for clinical consultation frequency (6- or 12-monthly) (S5 and S7 Figs).

**Fig 5:**
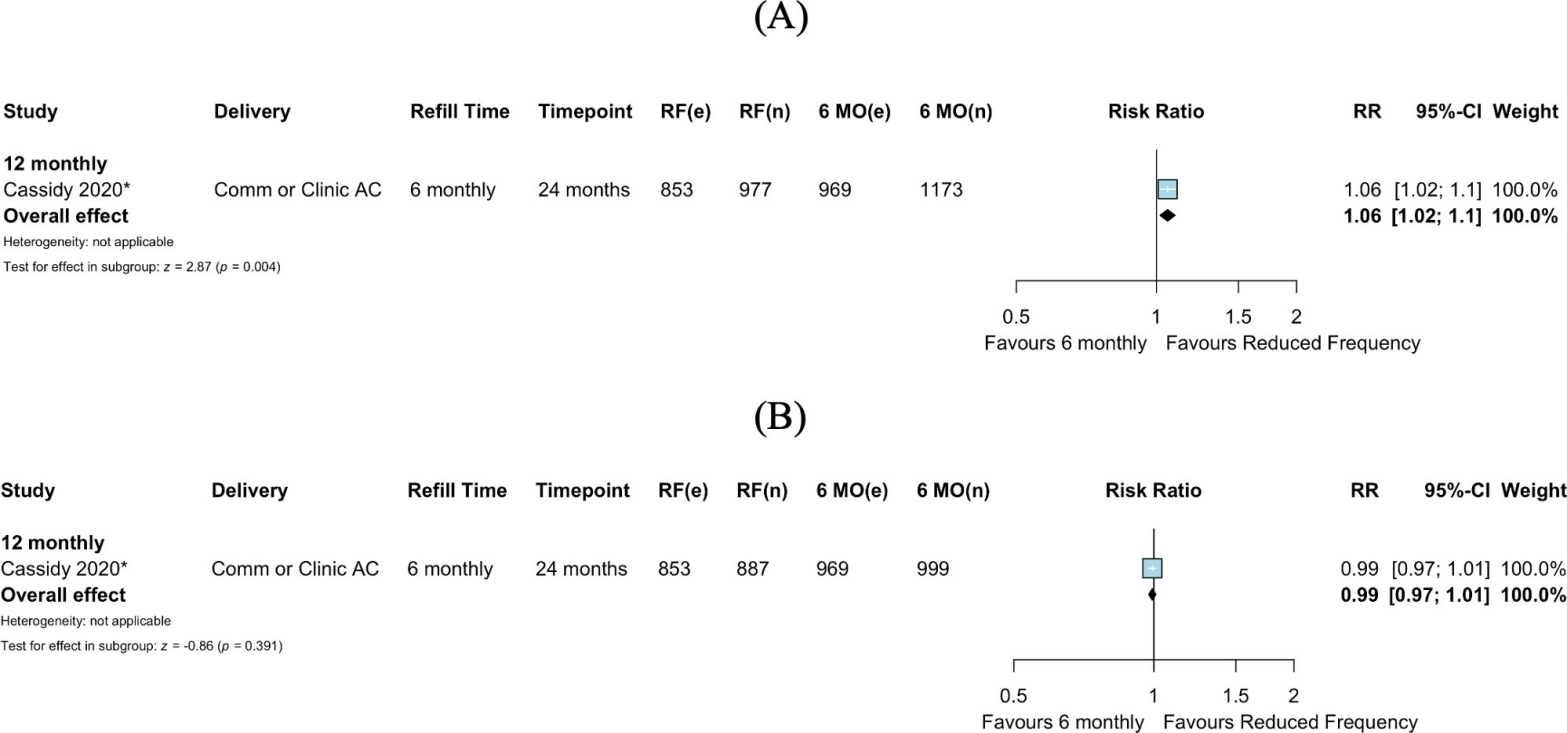
Viral suppression: Reduced frequency versus 6-monthly clinical consultations. (A) Among those randomized. (B) Among those with viral load testing. *Cluster-adjusted RR. 6 MO, 6-monthly; AC, adherence club; Comm, community; e, number of events; n, number of participants; RF, reduced frequency; RR, risk ratio.

In the single study comparing 12-monthly to 6-monthly clinical consultations, among all individuals randomized, viral suppression was higher in the arm with 12-monthly versus 6-monthly clinical consultations (RR 1.06, 95% CI 1.02–1.10, *p =* 0.004) (Fig 5A). Among those who received viral load testing, there was no difference in viral suppression for 12-monthly compared to 6-monthly clinical consultations (RR 0.99, 95% CI 0.97–1.01, *p =* 0.391) (Fig 5B).

#### Mortality

**Fig 6:**
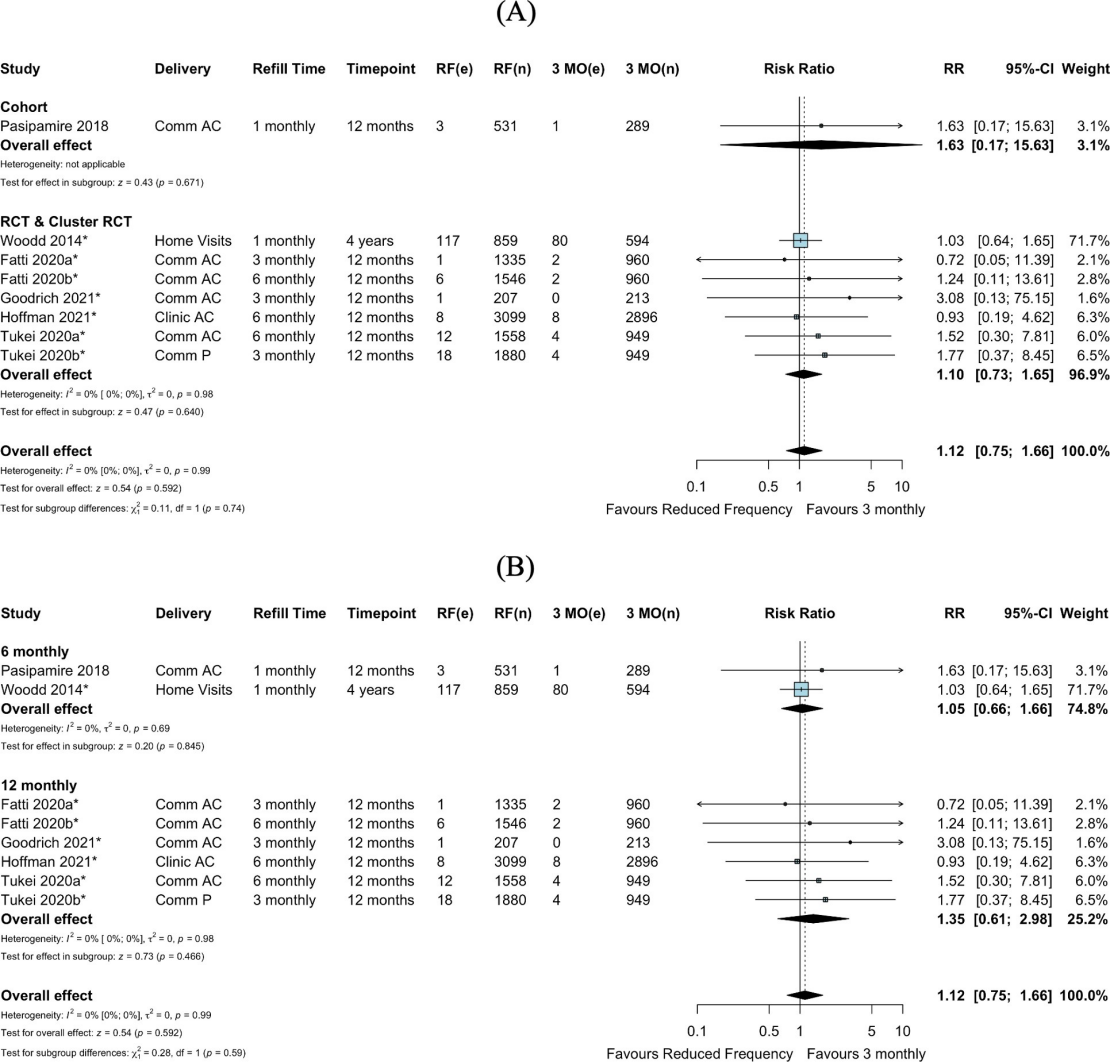
Mortality: Reduced frequency versus 3-monthly clinical consultations among randomized or enrolled individuals. (A) By study design. (B) By clinical consultation frequency. *Cluster-adjusted RR. 3 MO, 3-monthly; AC, adherence club; Comm, community; e, number of events; n, number of participants; P, pharmacy; RCT, randomized controlled trial; RF, reduced frequency; RR, risk ratio.

**Fig 7:**
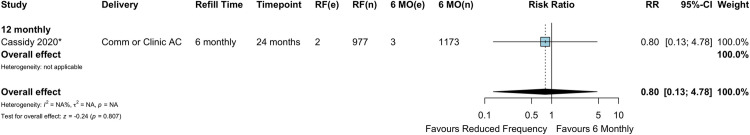
Mortality: Reduced frequency versus 6-monthly clinical consultations. *Cluster-adjusted RR. 6 MO, 6-monthly; AC, adherence club; Comm, community; e, number of events; n, number of participants; RF, reduced frequency; RR, risk ratio.

Seven comparative studies (9 comparisons), including 1 cohort study and 6 RCTs, contributed to the mortality meta-analysis. There was no evidence of a difference in mortality between reduced clinical consultations and 3-monthly consultations among these studies (overall RR 1.12, 95% CI 0.75–1.66, *p =* 0.592) (Fig 6A). This was consistent for 6-monthly clinical consultations and 12-monthly clinical consultations compared to 3-monthly consultations, though these estimates have wide confidence intervals due to the small numbers of events (Fig 6B). There was also no evidence of a difference in mortality when comparing further extended intervals (12-monthly) to 6-monthly clinical consultations (RR 0.80, 95% CI 0.13–4.78, *p =* 0.807) (Fig 7), though this comparison consists of only 1 study [25].

### Reduced ART refill dispensing frequency

#### Retention in care

**Fig 8:**
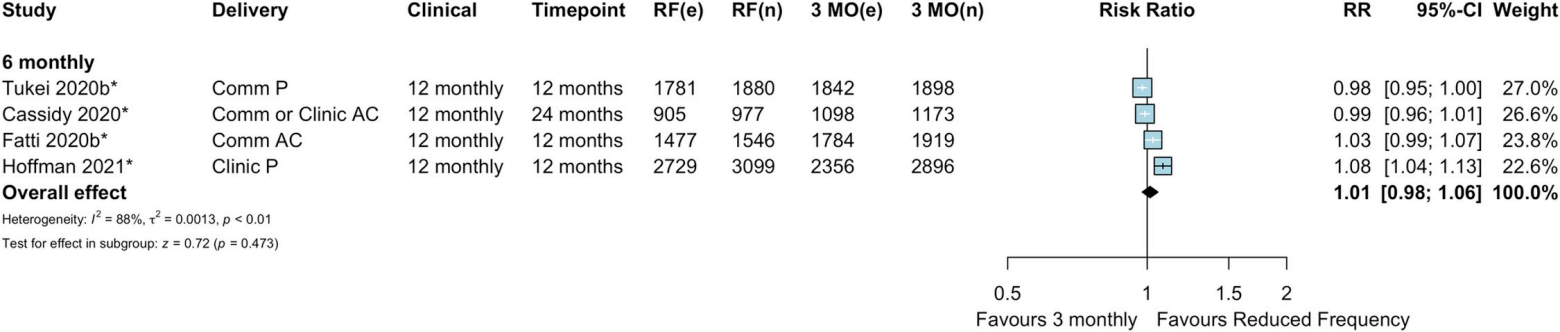
Retention in care: Reduced frequency versus 3-monthly antiviral treatment refills. *Cluster-adjusted RR. 3 MO, 3-monthly; AC, adherence club; Comm, community; e, number of events; n, number of participants; RF, reduced frequency; RR, risk ratio.

Among the 4 studies (all cluster RCTs) investigating reduced ART refill frequency, there appeared to be no difference in retention in care between increased (6 month) refill intervals and 3-monthly refill frequency (RR 1.01, 95% CI 0.98–1.06, *p =* 0.473) (Fig 8). No studies assessed ART refills at intervals greater than 6 months.

#### Viral suppression

**Fig 9:**
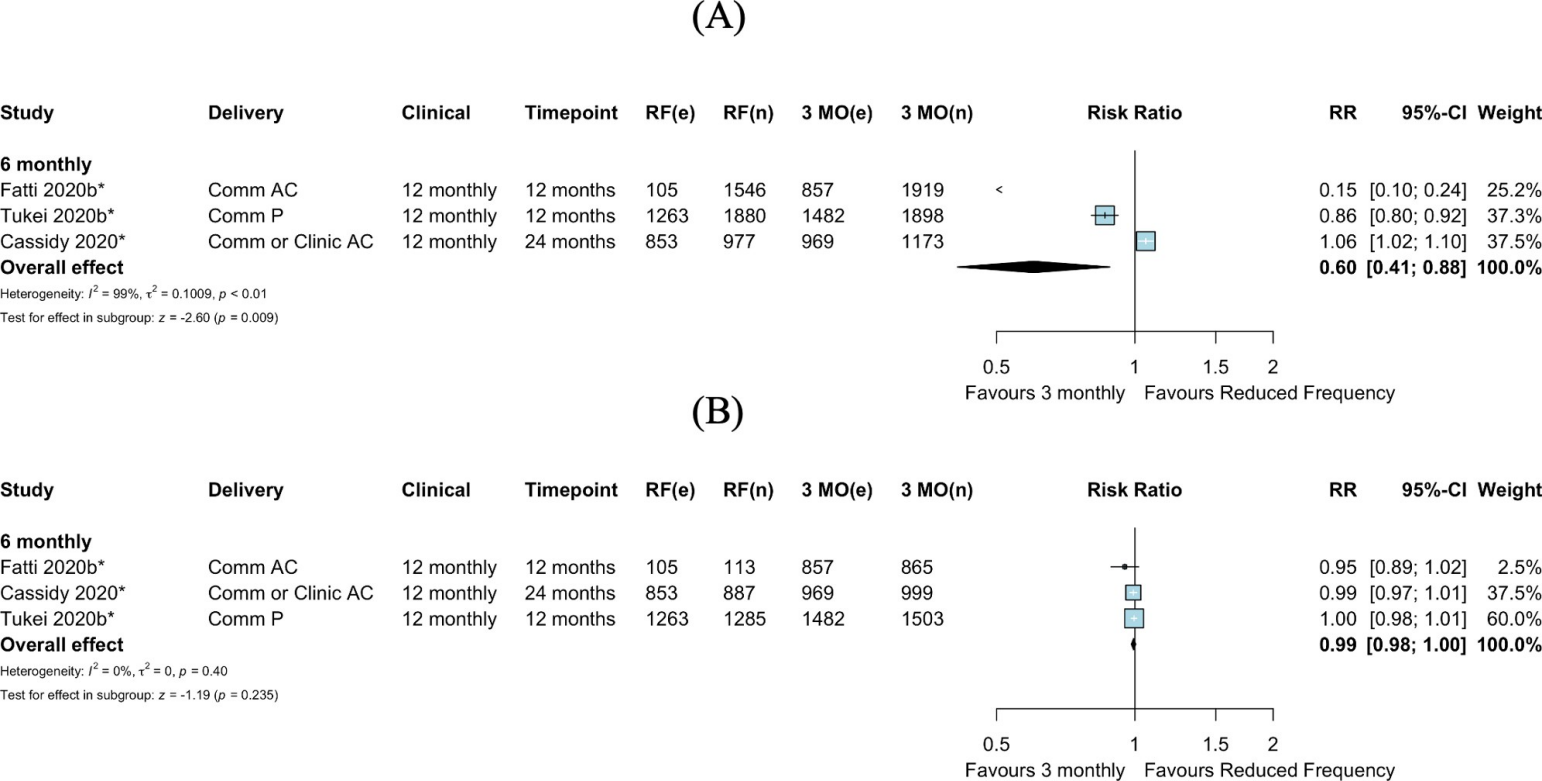
Viral suppression: Reduced frequency versus 3-monthly antiviral treatment refills, by study design. (A) Among those randomized. (B) Among those with viral load testing. *Cluster-adjusted RR. 3 MO, 3-monthly; AC, adherence club; Comm, community; e, number of events; n, number of participants; P, pharmacy; RF, reduced frequency; RR, risk ratio.

Among all PLWH enrolled in the 3 RCTs comparing reduced (6-monthly) ART dispensing frequency to 3-monthly dispensing, viral suppression appeared better among those in the 3-monthly dispensing arms (RR 0.60, 95% CI 0.41–0.88, *p =* 0.009) (Fig 9A). This comparison had substantial statistical heterogeneity, influenced by 2 studies with under-ascertainment of viral load in intervention arms [27,38] (S2 Table). The available case analysis, including only PLWH who received viral load testing, showed no difference between intervention arms for 6-monthly refills compared to 3-monthly refills (RR 0.99, 95% CI 0.98–1.00, *p =* 0.235) (Fig 9B).

#### Mortality

**Fig 10:**
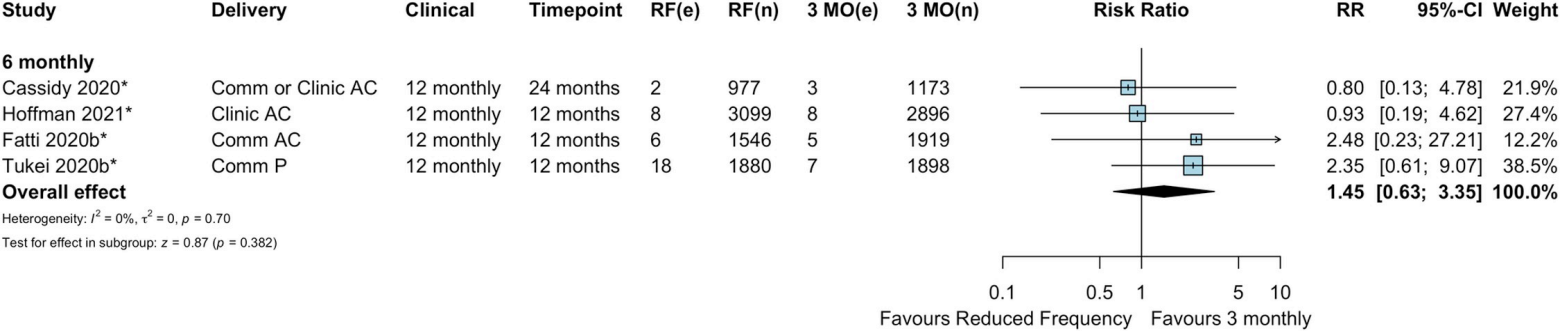
Mortality: Reduced frequency versus 3-monthly refills. *Cluster-adjusted RR. 3 MO, 3-monthly; AC, adherence club; Comm, community; e, number of events; n, number of participants; P, pharmacy; RF, reduced frequency; RR, risk ratio.

In the 4 studies (all RCTs) comparing 6-monthly refill frequency to 3-monthly refills, there was no evidence of a difference in mortality between reduced (6-monthly) ART dispensing frequency and 3-monthly refills (RR 1.45, 95% CI 0.63–3.35, *p =* 0.382) (Fig 10).

### Certainty of evidence (GRADE)

The certainty of the evidence (a combined assessment of strength of association, methodological quality, heterogeneity, and external validity) for the pooled data for the primary outcomes of retention, viral suppression, and mortality was assessed as very low to moderate quality (Tables 6 and 7). Effect estimates were downgraded due to high risk of bias in the contributing studies and heterogeneity in the contributing effect estimates. For clinical visit frequency comparisons, the certainty of evidence for retention in care was ranked overall as moderate, and viral suppression and mortality were rated as having very low quality evidence, largely due to imprecision and/or high risk of bias for contributing studies. Regarding the overall estimates for ART refill dispensing frequency comparisons, the outcome of retention in care was ranked as having moderate certainty, while viral suppression was ranked as having very low certainty, and mortality was ranked as having low certainty, also due to high risk of bias and imprecision.

**Table 6:** Review evidence certainty assessment (GRADE): Reduced clinical appointment frequency (6- or 12-monthly) versus 3-monthly clinical appointments.

Number of studies	Certainty assessment						Number of patients with outcome/number of patients total (%)		Effect estimate: Risk ratio (95% CI)	Certainty
	Study design	Risk of bias	Inconsistency	Indirectness	Imprecision	Other considerations	Reduced clinic appointment frequency	3-monthly clinic appointments		
**Retention in care among all enrolled at longest time point: Any reduced frequency versus 3-monthly clinic appointment frequency**										
8	RCTs & observational studies	Serious^a^	Serious^b^	Not serious	Not serious	None	11,526/12,638 (91.2%)	8,454/9,623 (87.9%)	1.01 (0.97 to 1.04)	⨁⨁◯◯ Low
**Retention in care among all enrolled at longest time point: RCTs**										
6	RCTs	Not serious	Serious^b^	Not serious	Not serious	None	9,892/10,759 (91.9%)	6,932/7,815 (88.7%)	1.00 (0.95 to 1.04)	⨁⨁⨁◯ Moderate
**Retention in care among all enrolled at longest time point: Cohort studies**										
3	Observational studies	Serious^c^	Not serious	Not serious	Not serious	None	1,634/1,879 (87.0%)	1,522/1,808 (84.2%)	1.02 (0.97 to 1.09)	⨁◯◯◯ Very low
**Retention in care among all enrolled at longest time point: 6-monthly versus 3-monthly clinic appointment frequency**										
4	RCTs & observational studies	Serious^a^	Not serious	Not serious	Not serious	None	2,602/3,013 (86.4%)	2,262/2,696 (83.9%)	1.03 (0.98 to 1.08)	⨁⨁⨁◯ Moderate
**Retention in care among all enrolled at longest time point: 12-monthly versus 3-monthly clinic appointment frequency**										
4	RCTs	Not serious	Serious^b^	Not serious	Not serious	None	8,924/9,625 (92.7%)	6,192/6,927 (89.3%)	0.99 (0.94 to 1.04)	⨁⨁⨁◯ Moderate
**Viral suppression among all enrolled at longest time point: RCTs**										
4	RCTs	Very serious^d^	Serious^b^	Not serious	Not serious	None	3,424/6,801 (50.3%)	2,777/4,325 (64.2%)	0.74 (0.59 to 0.94)	⨁◯◯◯ Very low
**Viral suppression among all enrolled at longest time point: Cohort studies**										
2	Observational studies	Not serious	Serious^b^	Not serious	Not serious	None	1,941/2,345 (82.8%)	2,934/6,729 (43.6%)	1.40 (0.95 to 2.08)	⨁◯◯◯ Very low
**Viral suppression among all who received viral load testing at longest time point: RCTs**										
4	RCTs	Not serious	Serious^b^	Not serious	Not serious	None	3,424/3,489 (98.1%)	2,777/2,826 (98.3%)	1.00 (0.92 to 1.08)	⨁⨁⨁◯ Moderate
**Viral suppression among all who received viral load testing at longest time point: Cohort studies**										
2	Observational studies	Not serious	Serious^b^	Not serious	Not serious	None	1,941/2,295 (84.6%)	2,934/2,962 (99.1%)	1.44 (1.24 to 1.66)	⨁◯◯◯ Very low
**Mortality among all enrolled at longest time point: Any reduced frequency versus 3-monthly clinic appointment frequency**										
6	RCTs & observational studies	Serious^a^	Not serious	Not serious	Very serious^e^	None	166/11,015 (1.5%)	101/7,810 (1.3%)	1.12 (0.75 to 1.66)	⨁◯◯◯ Very low
**Mortality among all enrolled at longest time point: RCTs**										
5	RCTs	Serious^d^	Not serious	Not serious	Very serious^e^	None	163/10,484 (1.6%)	100/7,521 (1.3%)	1.10 (0.73 to 1.65)	⨁⨁◯◯ Low
**Mortality among all enrolled at longest time point: Cohort studies**										
1	Observational studies	Serious^f^	Not serious	Not serious	Very serious^e^	None	3/531 (0.6%)	1/289 (0.3%)	1.63 (0.17 to 15.63)	⨁◯◯◯ Very low
**Mortality among all enrolled at longest time point: 6-monthly versus 3-monthly clinic appointment frequency**										
2	RCTs & observational studies	Serious^d^	Not serious	Not serious	Very serious^e^	None	120/1,390 (8.6%)	81/883 (9.2%)	1.05 (0.66 to 1.66)	⨁◯◯◯ Very low
**Mortality among all enrolled at longest time point: 12-monthly versus 3-monthly clinic appointment frequency**										
4	RCTs	Not serious	Not serious	Not serious	Very serious^e^	None	46/9,625 (0.5%)	20/6,927 (0.3%)	1.35 (0.61 to 2.98)	⨁⨁◯◯ Low

CI, confidence interval; RCT, randomized controlled trial. Explanations

^a^Combination of cohort and RCT data. In addition, contributing observational study was ranked as poor quality.

^b^Statistical heterogeneity.

^c^Variable study quality.

^d^Studies with high risk of bias and/or some concerns of bias contribute substantially to estimate.

^e^Downgraded due to very wide confidence intervals including benefit and harm.

^f^Estimate consists of only 1 study.

**Table 7:** Review evidence certainty assessment (GRADE): Reduced ART dispensing (6-monthly) versus 3-monthly ART dispensing.

Number of studies	Certainty assessment						Number of patients with outcome/number of patients total (%)		Effect estimate: Risk ratio (95% CI)	Certainty
	Study design	Risk of bias	Inconsistency	Indirectness	Imprecision	Other considerations	Reduced dispensing frequency	3-monthly dispensing frequency		
**Retention in care among all enrolled at longest time point**										
4	RCTs	Not serious^a^	Serious^b^	Not serious	Not serious	None	6,892/7,502 (91.9%)	7,080/7,886 (89.8%)	1.02 (0.97 to 1.06)	⨁⨁⨁◯ Moderate
**Viral suppression among all enrolled at longest time point**										
3	RCTs	Very serious^c^	Serious^b^	Not serious	Not serious	None	2,221/4,403 (50.4%)	3,308/4,990 (66.3%)	0.60 (0.41 to 0.88)	⨁◯◯◯ Very low
**Viral suppression among all who received viral load testing at longest time point**										
3	RCTs	Serious^d^	Not serious	Not serious	Not serious	None	2,221/2,285 (97.2%)	3,308/3,367 (98.2%)	0.99 (0.98 to 1.00)	⨁⨁⨁◯ Moderate
**Mortality among all enrolled at longest time point**										
4	RCTs	Not serious^a^	Not serious	Not serious	Very serious^e^	None	34/7,502 (0.5%)	23/7,886 (0.3%)	1.45 (0.63 to 3.35)	⨁⨁◯◯ Low

CI, confidence interval; RCT, randomized controlled trial.

^a^Four RCTs—3 with low risk of bias and 1 with some concerns.

^b^Marked statistical heterogeneity.

^c^Three RCTs—1 with some concerns and 2 with high risk of bias.

^d^Three RCTs—2 with low risk of bias and 1 with some concerns.

^e^Very few events and wide confidence intervals.

These evidence rankings contribute to statements and assumptions that can be made about the evidence contributing to this review. The very low quality evidence for viral load and mortality lead to final assessments of insufficient evidence to draw meaningful conclusions for these outcomes.

## Discussion

In this systematic review we found among the 10 included studies (6 RCTs, 3 observational studies, and 1 study contributing both observational and RCT data)—with 15 study arms with 33,599 adults in 8 countries in sub-Saharan Africa—that reduced frequency of clinical consultations and ART dispensing appeared to have comparable HIV treatment outcomes to 3-monthly clinical or dispensing visits. For reduced frequency clinical consultations, there was no evidence of a difference in retention in care, when comparing reduced frequency (i.e., 6- or 12-monthly) clinical consultations to 3-monthly consultation visits. For clinical consultations, viral load results were inconsistent, and it was not possible to discern the effect of reduced clinical consultation frequency on viral suppression due to marked under-ascertainment of viral load in reduced frequency intervention arms. Similarly, conclusions could not be drawn on the effects on mortality, due to the overall small number of events and very low quality evidence. A single study that compared 12-monthly to 6-monthly clinical consultations showed similar retention in care and viral suppression between study arms. When comparing 6-monthly to 3-monthly ART dispensing frequency, there appeared to be little to no difference in retention in care. For ART refill frequency, evidence quality ratings for viral suppression and mortality were similarly very low; it was therefore not possible to draw conclusions for these outcomes.

Visit frequency was reduced through a variety of implementation strategies: In most cases clinical consultations occurred at the health facility, and ART dispensing was facilitated through adherence clubs at the health facility or in the community, with individual club members, lay staff, or nurses distributing ART. Other community ART delivery strategies included distribution at community venues, private pharmacies, or mobile health units, or directly in the homes of PLWH, though there were relatively few studies to compare across delivery strategies. Overall, included studies were highly pragmatic. There was, however, marked heterogeneity of effects, study designs, risk of bias, implementation strategies, and outcome measurement time points—this contributed to the low-certainty evidence ratings for several outcomes. The definition of the established-on-ART patient population varied by study; however, no studies included data on children or key population groups, or were from outside of the sub-Saharan African region.

While we found overall little difference in clinical outcomes for reduced visit frequency, there are other potential benefits of reduced visits, including decongestion of health facilities, reduced provider workload, prioritization of care for new or clinically unstable PLWH, and reduced transmission of COVID-19 in health centers [5,13,41–44]. Reducing visit frequency has been reported to be one of the easiest DSD models to implement and aligns strongly with the care preferences of PLWH by reducing the economic costs of attending frequent appointments, reducing stigma, and allowing PLWH to normalize HIV [3,4,10,11,45,46]. HIV services, however, need to remain flexible enough to accommodate return to facilities for those who opt back into standard care or when clinical requirements change [27]. Further research is needed to develop strategies that allow for transition between models of care and provide psychosocial support between extended visits (e.g., virtual visits or group models) [3,47]. As many countries, in response to COVID-19, have expanded multi-month dispensing for patients who have not previously been considered established on ART [12], it will be essential to explore outcomes in those less “established” on ART, as well as to develop strategies to align and integrate non-communicable disease care with these models and to identify optimum models of care for key populations, other regions, and children, to ensure the utility of these models for all PLWH [5,48].

Ongoing successful scale-up of multi-month scripting and sustainability will depend on well-functioning drug supply chains. To date, 3-monthly ART dispensing visits and 6-monthly clinical consultations have been widely adopted in LMICs, and the COVID-19 epidemic has accelerated the adoption of even longer intervals, with 11 countries providing 6-monthly ART refills and 6 countries providing 12-monthly clinical consults as of June 2021 [12,49–51]. The reliability of supply chains to maintain multi-month dispensing remains a concern, however, with drug stock-outs common, particularly in the sub-Saharan African region [4,52]. At this time, local drug supply chains and pharmacy capacity should be robust to ensure that PLWH do not experience barriers to obtaining at minimum 3-monthly refills [53,54].

In addition to ensuring adequate ART supply, incorporating well-functioning treatment monitoring strategies into differentiated models of care will be crucial. While there were too few studies within subgroups to compare outcomes by delivery strategy, reducing facility visits reduces opportunities for viral load measurement at centralized locations, and viral load monitoring, in particular, appeared to be a challenge in treatment arms providing primarily community-based services with infrequent facility visits. Strengthening facility-based laboratory systems as well as establishing reliable decentralized viral load monitoring strategies (e.g., point-of-care or community-based sample collection) represent further areas for investigation to support reduced clinical and ART dispensing visit frequency [55–57].

### Limitations and strengths

This synthesis was strengthened by inclusion of a wide range of pragmatic trial data and programmatic observational data providing real-world insights into the effect of reducing dispensing and clinical visit intervals. There were, however, also several limitations of the data included in the review. First, we acknowledge that pooling heterogenous studies cannot generate one true effect estimate relevant to all contexts; however, such syntheses can give insights into the broader question of whether an intervention results in benefit or harm, which was the overarching goal of this synthesis. Second, there was a lack of evidence for children, key populations, and LMICs outside of sub-Saharan Africa, limiting the generalizability of the findings. Third, few studies contributed to comparisons of 12- versus 6-monthly clinical consultations, and therefore no firm conclusions could be generated on the relative effect of such consultation intervals. Fourth, there were insufficient data to compare and stratify by reduced visit interval implementation strategies. Lastly, there were no data on how increasing visit intervals impacts management of other comorbid illnesses such as diabetes and hypertension.

## Conclusion

Based on data from this synthesis, extending clinical consultation intervals beyond 3 months and ART dispensing intervals to 6 months likely results in similar retention in care compared to 3-monthly intervals, with uncertain effects on mortality and viral suppression. As countries shift toward 6-monthly clinical consultations and extended ART dispensation intervals, research should identify which delivery strategies are most efficient in accommodating both patient preferences and pragmatic concerns regarding cost and logistical health system capabilities. Ongoing monitoring of emerging evidence on the scale-up of reduced visit interval strategies will be critical to inform future HIV service delivery guidelines.

## Supporting information

S1 Appendix Deviations from protocol listed in PROSPERO. (DOCX)

S2 Appendix Search terms. (DOCX)

S1 Fig Retention in care: Reduced frequency versus 3-monthly clinical consultations, by delivery strategy. Fox 2019a and Fox 2019b were separated into an RCT and cohort design, respectively, based on the analysis described by the authors, where randomization was not preserved in the intervention arm in Fox 2019b. *Cluster-adjusted RR. 3 MO, 3-monthly; AC, adherence club; Comm, community; e, number of events; n, number of participants; ND, not described; P, pharmacy; RF, reduced frequency.
(TIF)

S2 Fig Retention in care: Reduced frequency versus 3-monthly clinical consultations, by risk of bias assessment. Fox 2019a and Fox 2019b were separated into an RCT and cohort design, respectively, based on the analysis described by the authors, where randomization was not preserved in the intervention arm in Fox 2019b. *Cluster-adjusted RR. 3 MO, 3-monthly; AC, adherence club; Comm, community; e, number of events; n, number of participants; ND, not described; P, pharmacy; RF, reduced frequency.
(TIF)

S3 Fig Retention in care: Reduced frequency versus 3-monthly clinical consultations, by time on ART for established-on-ART patient population. Fox 2019a and Fox 2019b were separated into an RCT and cohort design, respectively, based on the analysis described by the authors, where randomization was not preserved in the intervention arm in Fox 2019b. *Cluster-adjusted RR. 3 MO, 3-monthly; AC, adherence club; Comm, community; e, number of events; n, number of participants; ND, not described; P, pharmacy; RF, reduced frequency.
(TIF)

S4 Fig Viral suppression among those enrolled: Reduced frequency versus 3-monthly clinical consultations, by clinical consultation frequency. Fox 2019a and Fox 2019b were separated into an RCT and cohort design, respectively, based on the analysis described by the authors, where randomization was not preserved in the intervention arm in Fox 2019b. *Cluster-adjusted RR. 3 MO, 3-monthly; AC, adherence club; Comm, community; e, number of events; n, number of participants; ND, not described; P, pharmacy; RF, reduced frequency.
(TIF)

S5 Fig Viral suppression among those with viral load testing: Reduced frequency versus 3-monthly clinical consultations, by clinical consultation frequency. Fox 2019a and Fox 2019b were separated into an RCT and cohort design, respectively, based on the analysis described by the authors, where randomization was not preserved in the intervention arm in Fox 2019b. *Cluster-adjusted RR. 3 MO, 3-monthly; AC, adherence club; Comm, community; e, number of events; n, number of participants; RF, reduced frequency.
(TIF)

S6 Fig Viral suppression among those enrolled: Reduced frequency versus 3-monthly clinical consultations, by delivery strategy. Fox 2019a and Fox 2019b were separated into an RCT and cohort design, respectively, based on the analysis described by the authors, where randomization was not preserved in the intervention arm in Fox 2019b. *Cluster-adjusted RR. 3 MO, 3-monthly; AC, adherence club; Comm, community; e, number of events; n, number of participants; RF, reduced frequency.
(TIF)

S7 Fig Viral suppression among those with viral load testing: Reduced frequency versus 3-monthly clinical consultations, by delivery strategy. Fox 2019a and Fox 2019b were separated into an RCT and cohort design, respectively, based on the analysis described by the authors, where randomization was not preserved in the intervention arm in Fox 2019b. *Cluster-adjusted RR. 3 MO, 3-monthly; AC, adherence club; Comm, community; e, number of events; n, number of participants; RF, reduced frequency.
(TIF)

S1 PRISMA Checklist (DOCX)

S1 Table Outcome definitions by study. (DOCX)

S2 Table Ascertainment of viral suppression by study. (DOCX)

S3 Table Risk of bias. (DOCX)

## Abbreviations

ART: antiretroviral treatment
CI: confidence interval
DSD: differentiated service delivery
LMICs: low- and middle-income countries
PLWH: people living with HIV
RCT: randomized controlled trial
RR: risk ratio
WHO: World Health Organization
