# Supplementary material for: Evaluation of HIV treatment outcomes with reduced frequency of clinical encounters and antiretroviral treatment refills: A systematic review and meta-analysis
Source: PLoS Med. 2022 Mar 22;19(3):e1003959. doi: 10.1371/journal.pmed.1003959 (PMC8982898; doi:10.1371/journal.pmed.1003959)
Supplement: S1 Appendix — (DOCX) [file pmed.1003959.s001.docx]

**S1 Appendix. Deviations from protocol listed in PROSPERO**

The protocol is registered in PROSPERO (CRD42019128609). Originally, we planned to analyze lengthening the interval between any clinic encounters (both clinical consultations and/or ART dispensing) beyond the standard of care. This included standard of care arms of 1 monthly and 2 monthly. After preparing analysis and acknowledging current guidelines recommending 3 monthly refills and clinical consultations, we included only comparisons of reduced frequency beyond 3 monthly (6 and 12 monthly). We also analyzed reduced frequencies of clinical consultations and ART dispensing separately. We also intended on evaluating adherence outcomes, but due to lack of reporting in the included studies, we did not evaluate this outcome.
