# Supplementary material for: Evaluation of HIV treatment outcomes with reduced frequency of clinical encounters and antiretroviral treatment refills: A systematic review and meta-analysis
Source: PLoS Med. 2022 Mar 22;19(3):e1003959. doi: 10.1371/journal.pmed.1003959 (PMC8982898; doi:10.1371/journal.pmed.1003959)
Supplement: S2 Appendix — (DOCX) [file pmed.1003959.s002.docx]

**S2 Appendix. Search Terms**

Database – Ovid Medline- Searched 11/9/2021

Limiters: Publication – 01/01/2010-11/9/2021; English only, humans

| Action | Term |
| --- | --- |
| 1 | exp HIV/ or human immunodeficiency virus.mp |
| 2 | exp Acquired immunodeficiency syndrome/ or exp Hiv Infections/ or human immunodeficiency virus infected patient.mp |
| 3 | (HIV or "HIV AIDS" or "hiv infect*" or "aids virus" or "cd4" ).ab.kw.ti |
| 4 | ("hiv1" or "hiv2" or "hiv-1" or "hiv-2").ab,kw,ti. |
| 5 | ("human immunodeficiency virus" or "human immune-deficiency virus" or "human immunedeficiency virus" or "human immuno-deficiency virus").ab,kw,ti. |
| 6 | ("acquired immunodeficiency syndrome" or "acquired immuno-deficiency syndrome" or "acquired immunedeficiency syndrome" or "acquired immune-deficiency syndrome").ab,kw,ti |
| 7 | Or/1-6 |
| 8 | exp anti-retroviral agents/ or antiretroviral agent.mp |
| 9 | exp antiretroviral therapy, highly active/ or exp Anti-HIV agents/ or antiretroviral therapy.mp |
| 10 | exp receptors, ccr5/ |
| 11 | exp HIV Fusion Inhibitors/ or *Antiviral Agents/ |
| 12 | *HIV Integrase Inhibitor/ or *HIV Protease Inhibitor/ |
| 13 | ("antiretroviral agent*" or "anti hiv agent*" or "anti aids agent" or "anti hiv drug" or "anti aids drug" or "haart" or "art" or "antiretroviral*" or "anti-retroviral therapy" or "antiretroviral therapy" or "highly active antiretroviral therapy" or "antiretroviral treatment").ab,kw,ti. |
| 14 | Or/8-13 |
| 15 | Exp Ambulatory Care/ or ambulatory care.mp |
| 16 | Exp "Appointments and Schedules"/ |
| 17 | *Health Planning/ or *Community Health Planning/ |
| 18 | (visit* adj2 (interval* or monthly or frequency or spacing or pharmacy or clinic or clinical)).ab,kw.ti. |
| 19 | (appointment* adj2 (interval* or monthly or frequency or spacing or pharmacy or clinic or clinical)).ab,kw.ti. |
| 20 | ("follow up" adj2 (clinic OR visit OR appointment OR monthly)).ab,kw,ti. |
| 21 | ("medication dispensing" or "medicine dispensing" or "dispensing interval*" or "best interval*" or "monthly dispensing" or "multi-month" or "multi month" or "home delivery").ab,ti,kw. |
| 22 | (refill* adj2 (drug* or "pharmacy-only" or "ART" or antiretroviral or "fast track")).ab,kw.ti. |
| 23 | ((community OR 'community-based') adj2 ('points of distribution' OR 'points of care' OR 'distribution point*' OR pharmacy OR 'art group' OR 'art refill group' OR 'care group' OR model OR 'client-led')).ab,kw,ti. |
| 24 | ('differentiated care' or 'differentiated service delivery' or 'DSD' or 'differentiated service*' or 'differentiated ART delivery' or 'adherence club' or 'adherence group' or 'youth club' or 'family club' ).ab,kw.ti. |
| 25 | (appointment* or refill* or "quick pick-up" or "PODI" or "CDDP" or "CAG" or "CCLAD"):ab,ti,kw |
| 26 | Or/15-26 |
| 27 | 7 AND 14 AND 26 with limiters |

Database – Embase - Searched 11/9/2021

Limiters: Publication – 01/01/2010-11/9/2021; English only, humans

| Action | Term |
| --- | --- |
| 1 | 'Human immunodeficiency virus infection'/exp OR 'Human immunodeficiency virus'/exp OR 'Human immunodeficiency virus infected patient'/exp OR 'acquired immune deficiency syndrome'/exp with limiters |
| 2 | ('hiv':ti,ab,kw OR 'hiv infect*':ti,ab,kw OR 'hiv-1':ti,ab,kw OR 'hiv1':ti,ab,kw OR 'hiv-2':ti,ab,kw OR 'hiv2':ti,ab,kw OR 'hiv aids':ti,ab,kw OR 'aids virus':ti,ab,kw OR 'cd4':ti,ab,kw) with limiters |
| 3 | ('human immunodeficiency virus':ti,ab,kw OR 'human immunedificiency virus':ti,ab,kw OR 'human immuno-deficiency virus':ti,ab,kw OR 'human immune-deficiency virus':ti,ab,kw) with limiters |
| 4 | ('acquired immunodeficiency syndrome' or 'acquired immuno-deficiency syndrome' or 'acquired immunedeficiency syndrome' or 'acquired immune-deficiency syndrome'):ab,ti,kw with limiters |
| 5 | Or/1-4 |
| 6 | ('antiretrovirus agent'/exp OR 'antiretroviral therapy'/exp OR 'highly active antiretroviral therapy'/exp OR 'anti human immunodeficiency virus agent'/exp OR 'chemokine receptor ccr5 antagonist'/exp OR 'human immunodeficiency virus fusion inhibitor'/exp OR 'integrase inhibitor'/exp/mj OR 'human immunodeficiency virus proteinase inhibitor'/exp/mj) with limiters |
| 7 | ('antiretroviral agent*':ab,ti,kw OR 'anti hiv agent*':ab,ti,kw OR 'anti aids agent*':ab,ti,kw OR 'anti hiv drug*':ab,ti,kw OR 'anti aids drug*':ab,ti,kw OR 'haart':ab,ti,kw OR 'art':ab,ti,kw OR 'antiretroviral*':ab,ti,kw OR 'antiretroviral therapy':ab,ti,kw OR 'highly active antiretroviral therapy':ab,ti,kw OR 'antiretroviral treatment':ab,ti,kw) with limiters |
| 8 | Or/6-7 |
| 9 | ('hospital management'/exp/mj OR 'outpatient department'/exp OR 'ambulatory care'/exp OR 'health care planning'/exp/mj) with limiters |
| 10 | (('visit*' NEAR/2 (interval* or 'monthly' or 'frequency' or 'spacing' or 'pharmacy' or 'clinic' or 'clinical'):ab,ti,kw) with limiters |
| 11 | (('appointment*" NEAR/2 (interval*' or 'monthly' or 'frequency' or 'spacing' or 'pharmacy' or 'clinic' or 'clinical'):ab,ti,kw) with limiters |
| 12 | (('follow up' NEAR/2 ('clinic' OR 'visit' OR 'appointment' OR 'monthly'):ab,ti,kw) with limiters |
| 13 | (('medication dispensing' or 'medicine dispensing' or 'dispensing interval*' or 'best interval*' or 'monthly dispensing' or 'multi-month' or 'multi month' or 'home delivery'):ab,ti,kw) with limiters |
| 14 | ((refill* NEAR/2 ('drug*' or 'pharmacy-only' or 'ART' or 'antiretroviral' or 'fast track'):ab,ti,kw) with limiters |
| 15 | (((community OR 'community-based') NEAR/2 ('points of distribution' OR 'points of care' OR 'distribution point*' OR pharmacy OR 'art group' OR 'art refill group' OR 'care group' OR model OR 'client-led')):ab,ti,kw) |
| 16 | (('differentiated care' or 'differentiated service delivery' or 'DSD' or 'differentiated service*' or 'differentiated ART delivery' or 'adherence club' or 'adherence group' or 'youth club' or 'family club' ):ab,ti,kw) with limiters |
| 17 | ('appointment*' or refill* or 'quick pick-up' or 'PODI' or 'CDDP' or 'CAG' or 'CCLAD'):ab,ti,kw |
| 18 | Or/9-17 |
| 19 | 5 AND 8 AND 18 |

Database – Cochrane Library (includes CENTRAL and ICTRP) Searched 11/9/2021

Limiters: Publication – 01/01/2010-11/9/2021; English only, humans

| Action | Term |
| --- | --- |
| 1 | MeSH descriptor: [HIV] explode all trees |
| 2 | MeSH descriptor: [HIV infections] explode all trees |
| 3 | MeSH descriptor: [Acquired Immunodeficiency Syndrome] explode all trees |
| 4 | ('hiv':ti,ab,kw OR 'hiv infect*':ti,ab,kw OR 'hiv-1':ti,ab,kw OR 'hiv1':ti,ab,kw OR 'hiv-2':ti,ab,kw OR 'hiv2':ti,ab,kw OR 'hiv aids':ti,ab,kw OR 'aids virus':ti,ab,kw OR 'cd4':ti,ab,kw) |
| 5 | ("human immunodeficiency virus" or "human immune-deficiency virus" or "human immunedeficiency virus" or "human immuno-deficiency virus"):ab,kw,ti |
| 6 | ("acquired immunodeficiency syndrome" or "acquired immuno-deficiency syndrome" or "acquired immunedeficiency syndrome" or "acquired immune-deficiency syndrome"):ab,kw,ti |
| 7 | {OR #1-#6} |
| 8 | MeSH descriptor: [Anti-Retroviral Agents] explode all trees |
| 9 | MeSH descriptor: [Antiretroviral Therapy, Highly Active] explode all trees |
| 10 | MeSH descriptor: [Anti-HIV Agents] explode all trees |
| 11 | MeSH descriptor: [CCR5 Receptor Antagonists] explode all trees |
| 12 | MeSH descriptor: [HIV Fusion Inhibitors] explode all trees |
| 13 | MeSH descriptor: [HIV Integrase Inhibitors] explode all trees |
| 14 | MeSH descriptor: [HIV Protease Inhibitors] explode all trees |
| 15 | ('antiretroviral agent*':ab,ti,kw OR 'anti hiv agent*':ab,ti,kw OR 'anti aids agent*':ab,ti,kw OR 'anti hiv drug*':ab,ti,kw OR 'anti aids drug*':ab,ti,kw OR 'haart':ab,ti,kw OR 'art':ab,ti,kw OR 'antiretroviral*':ab,ti,kw OR 'antiretroviral therapy':ab,ti,kw OR 'highly active antiretroviral therapy':ab,ti,kw OR 'antiretroviral treatment':ab,ti,kw) |
| 16 | {OR #8-#15} |
| 17 | MeSH descriptor: [Appointments and Schedules] explode all trees |
| 18 | MeSH descriptor: [Ambulatory Care] explode all trees |
| 19 | MeSH descriptor: [Community Health Planning] explode all trees |
| 20 | visit* NEAR/2(interval* or monthly or frequency or spacing or pharmacy or clinic or clinical):ab,ti,kw |
| 21 | appointment* NEAR/2 (interval* or monthly or frequency or spacing or pharmacy or clinic or clinical):ab,ti.kw |
| 22 | 'follow up' NEAR/2 (clinic OR visit OR appointment OR monthly):ab,ti,kw |
| 23 | ('medication dispensing' or 'medicine dispensing' or 'dispensing interval*' or 'best interval*' or 'monthly dispensing' or 'multi-month' or 'multi month' or 'home delivery'):ab,ti,kw |
| 24 | refill* NEAR/2 (drug* or 'pharmacy-only' or 'ART' or antiretroviral or 'fast track'):ab,ti,kw |
| 25 | ((community OR 'community-based') NEAR/2 ('points of distribution' OR 'points of care' OR 'distribution point*' OR pharmacy OR 'art group' OR 'art refill group' OR 'care group' OR model OR 'client-led')):ab,ti,kw |
| 26 | ('differentiated care' or 'differentiated service delivery' or 'DSD' or 'differentiated service*' or 'differentiated ART delivery' or 'adherence club' or 'adherence group' or 'youth club' or 'family club' ):ab,ti,kw |
| 27 | (appointment* or refill* or 'quick pick-up' or 'PODI' or 'CDDP' or 'CAG' or 'CCLAD'):ab,ti,kw |
| 28 | {OR #17-#27} |
| 26 | #7 AND #16 AND #28 with limiters |
