# Supplementary figures and images for: Evaluation of HIV treatment outcomes with reduced frequency of clinical encounters and antiretroviral treatment refills: A systematic review and meta-analysis
Source: PLoS Med. 2022 Mar 22;19(3):e1003959. doi: 10.1371/journal.pmed.1003959 (PMC8982898; doi:10.1371/journal.pmed.1003959)

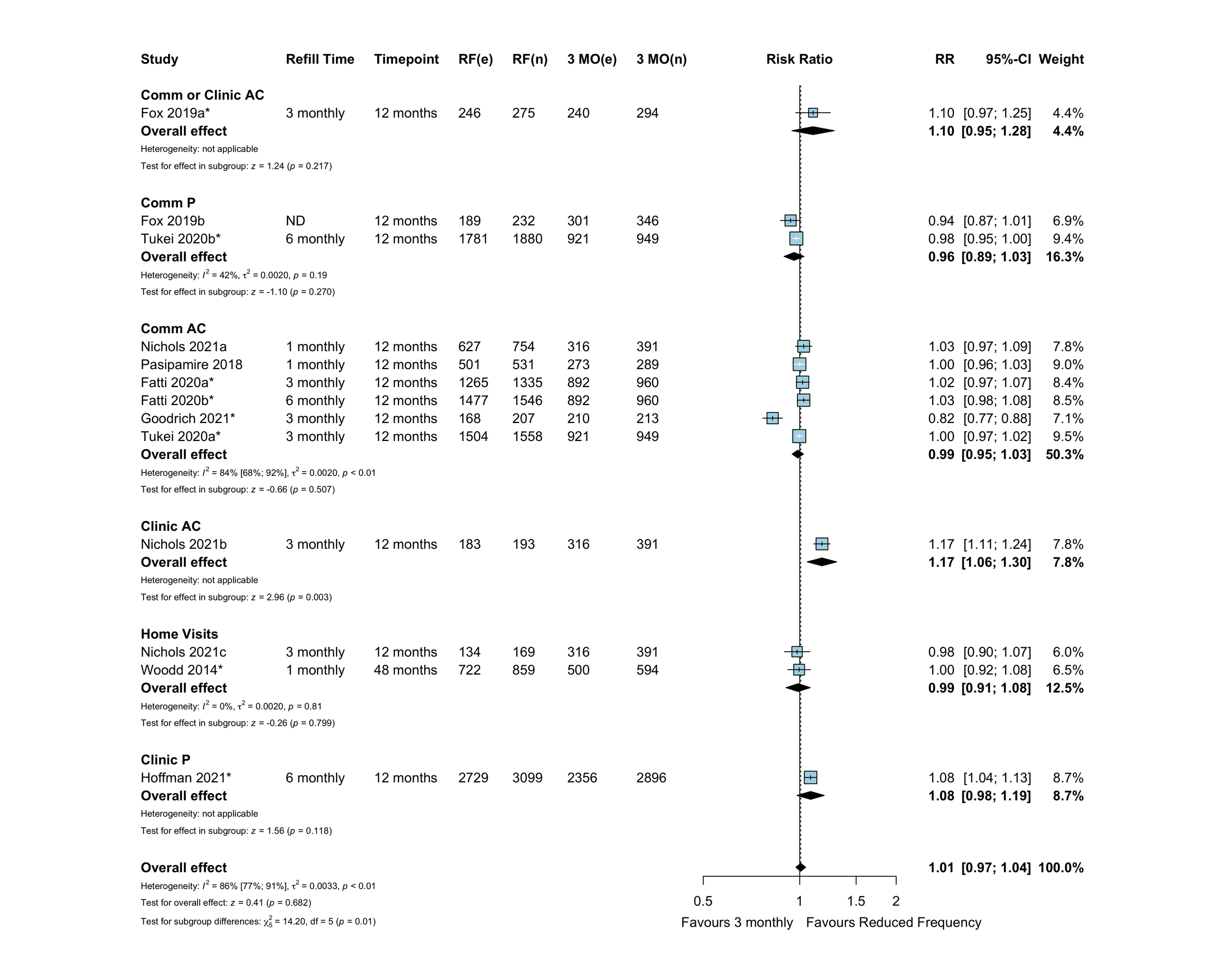

Supplement: S1 Fig — Fox 2019a and Fox 2019b were separated into an RCT and cohort design, respectively, based on the analysis described by the authors, where randomization was not preserved in the intervention arm in Fox 2019b. *Cluster-adjusted RR. 3 MO, 3-monthly; AC, adherence club; Comm, community; e, number of events; n, number of participants; ND, not described; P, pharmacy; RF, reduced frequency. (TIF) [file pmed.1003959.s003.tif]

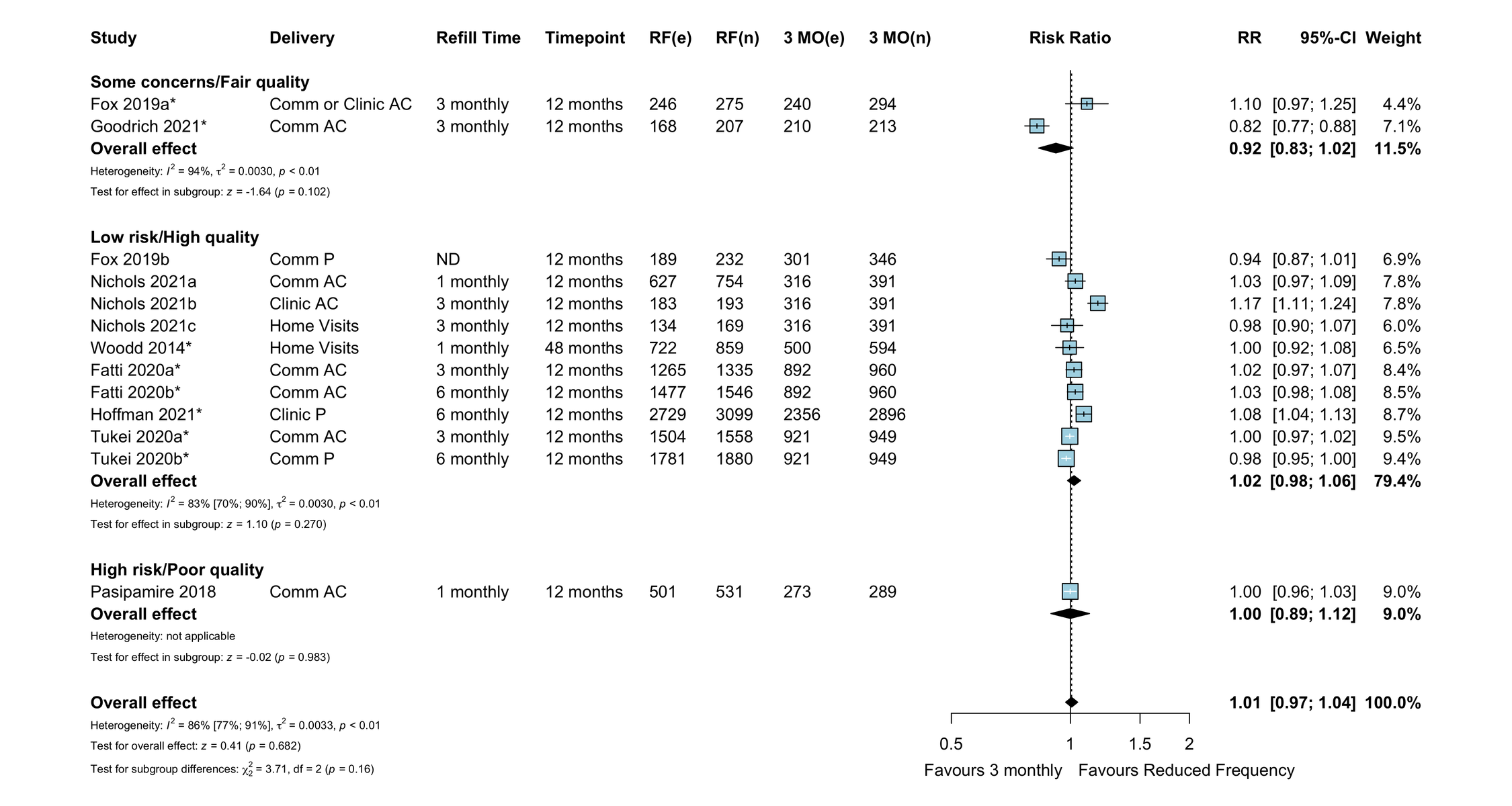

Supplement: S2 Fig — Fox 2019a and Fox 2019b were separated into an RCT and cohort design, respectively, based on the analysis described by the authors, where randomization was not preserved in the intervention arm in Fox 2019b. *Cluster-adjusted RR. 3 MO, 3-monthly; AC, adherence club; Comm, community; e, number of events; n, number of participants; ND, not described; P, pharmacy; RF, reduced frequency. (TIF) [file pmed.1003959.s004.tif]

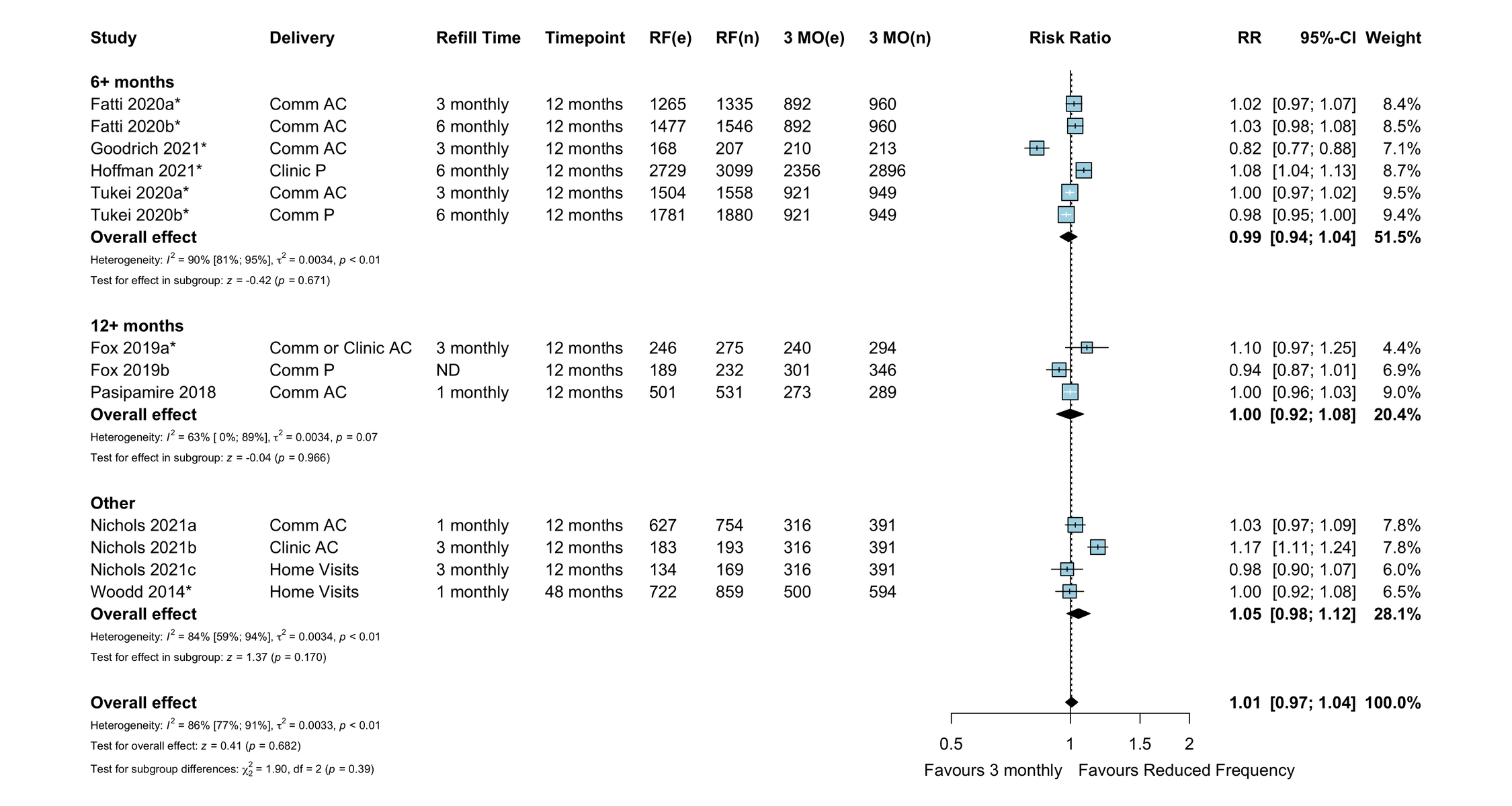

Supplement: S3 Fig — Fox 2019a and Fox 2019b were separated into an RCT and cohort design, respectively, based on the analysis described by the authors, where randomization was not preserved in the intervention arm in Fox 2019b. *Cluster-adjusted RR. 3 MO, 3-monthly; AC, adherence club; Comm, community; e, number of events; n, number of participants; ND, not described; P, pharmacy; RF, reduced frequency. (TIF) [file pmed.1003959.s005.tif]

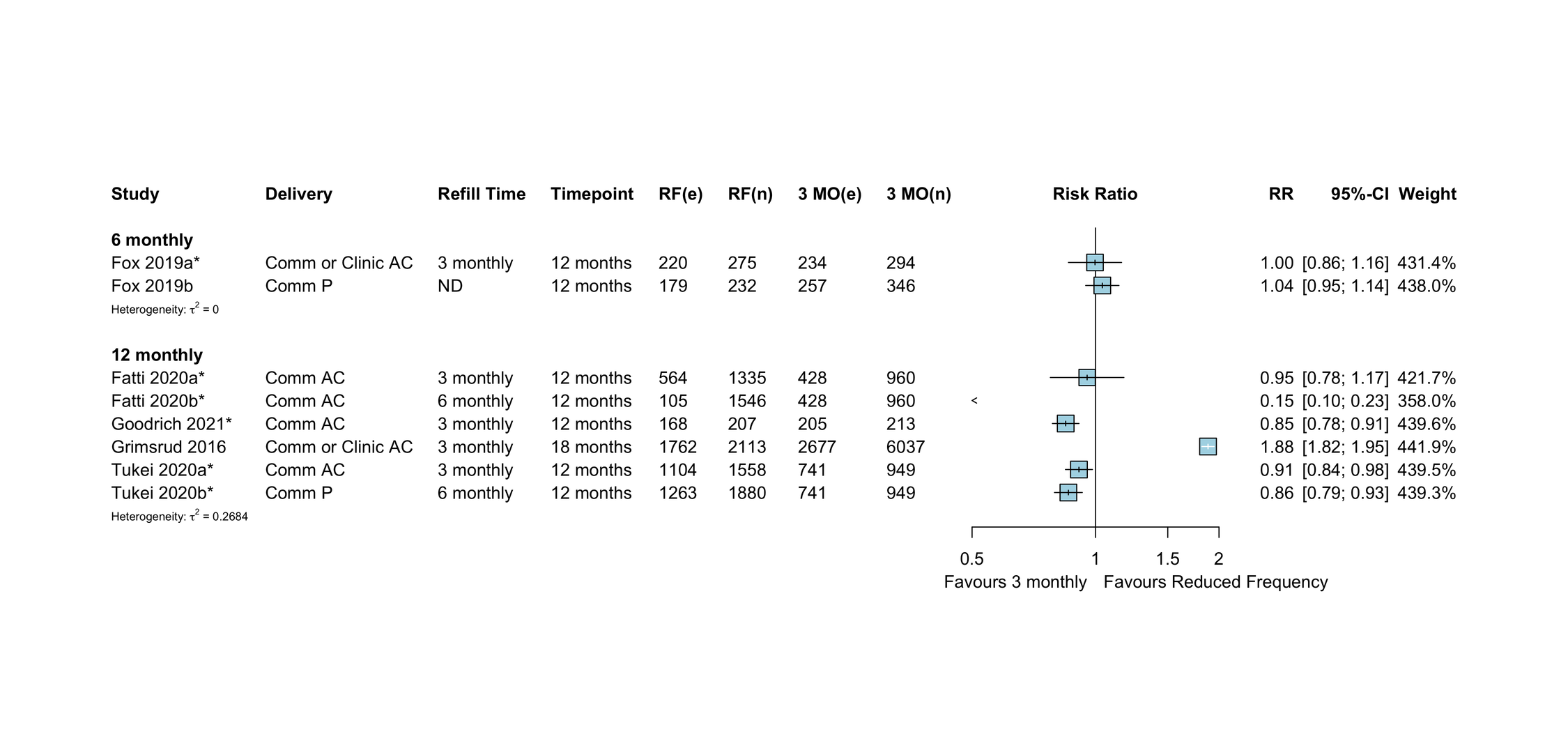

Supplement: S4 Fig — Fox 2019a and Fox 2019b were separated into an RCT and cohort design, respectively, based on the analysis described by the authors, where randomization was not preserved in the intervention arm in Fox 2019b. *Cluster-adjusted RR. 3 MO, 3-monthly; AC, adherence club; Comm, community; e, number of events; n, number of participants; ND, not described; P, pharmacy; RF, reduced frequency. (TIF) [file pmed.1003959.s006.tif]

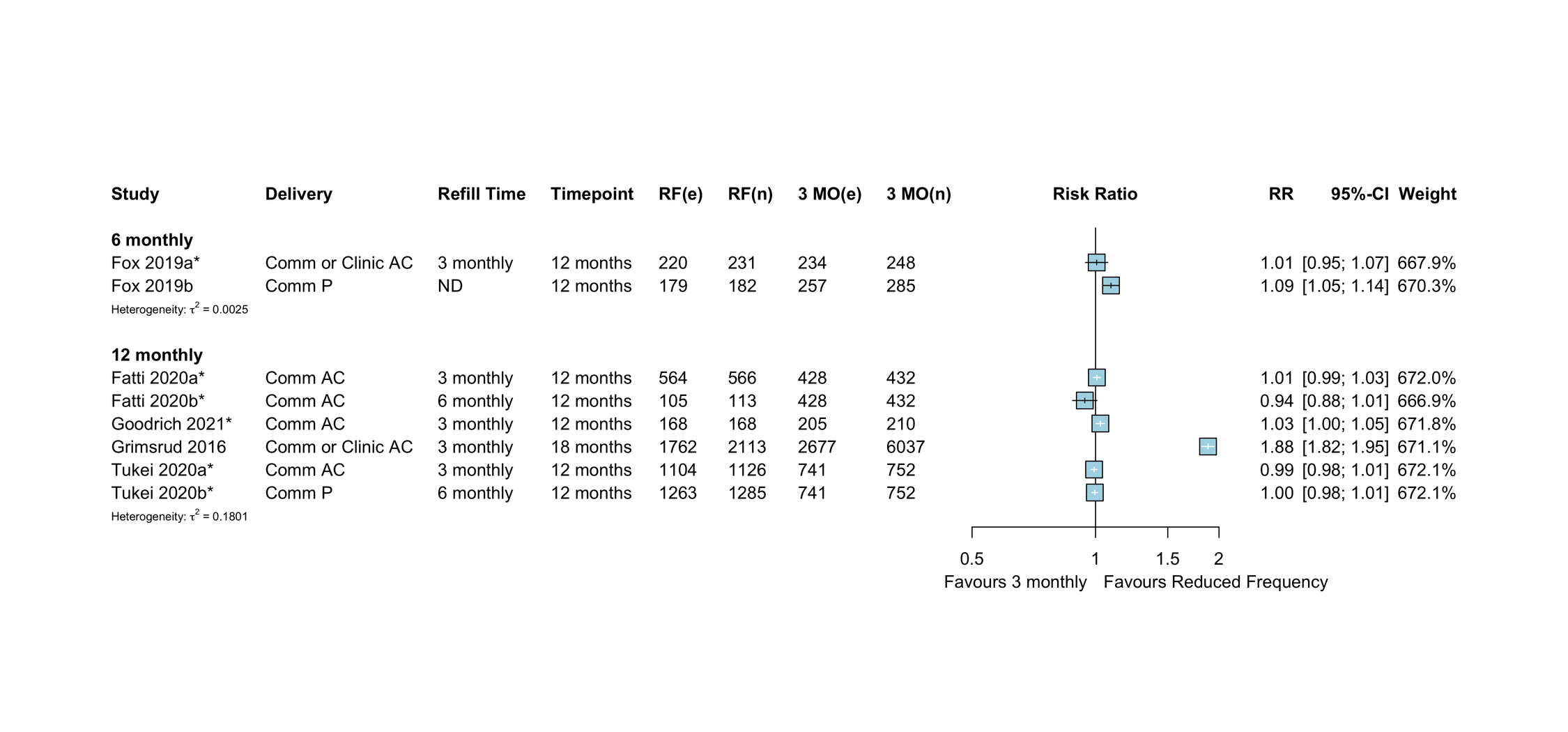

Supplement: S5 Fig — Fox 2019a and Fox 2019b were separated into an RCT and cohort design, respectively, based on the analysis described by the authors, where randomization was not preserved in the intervention arm in Fox 2019b. *Cluster-adjusted RR. 3 MO, 3-monthly; AC, adherence club; Comm, community; e, number of events; n, number of participants; RF, reduced frequency. (TIF) [file pmed.1003959.s007.tif]

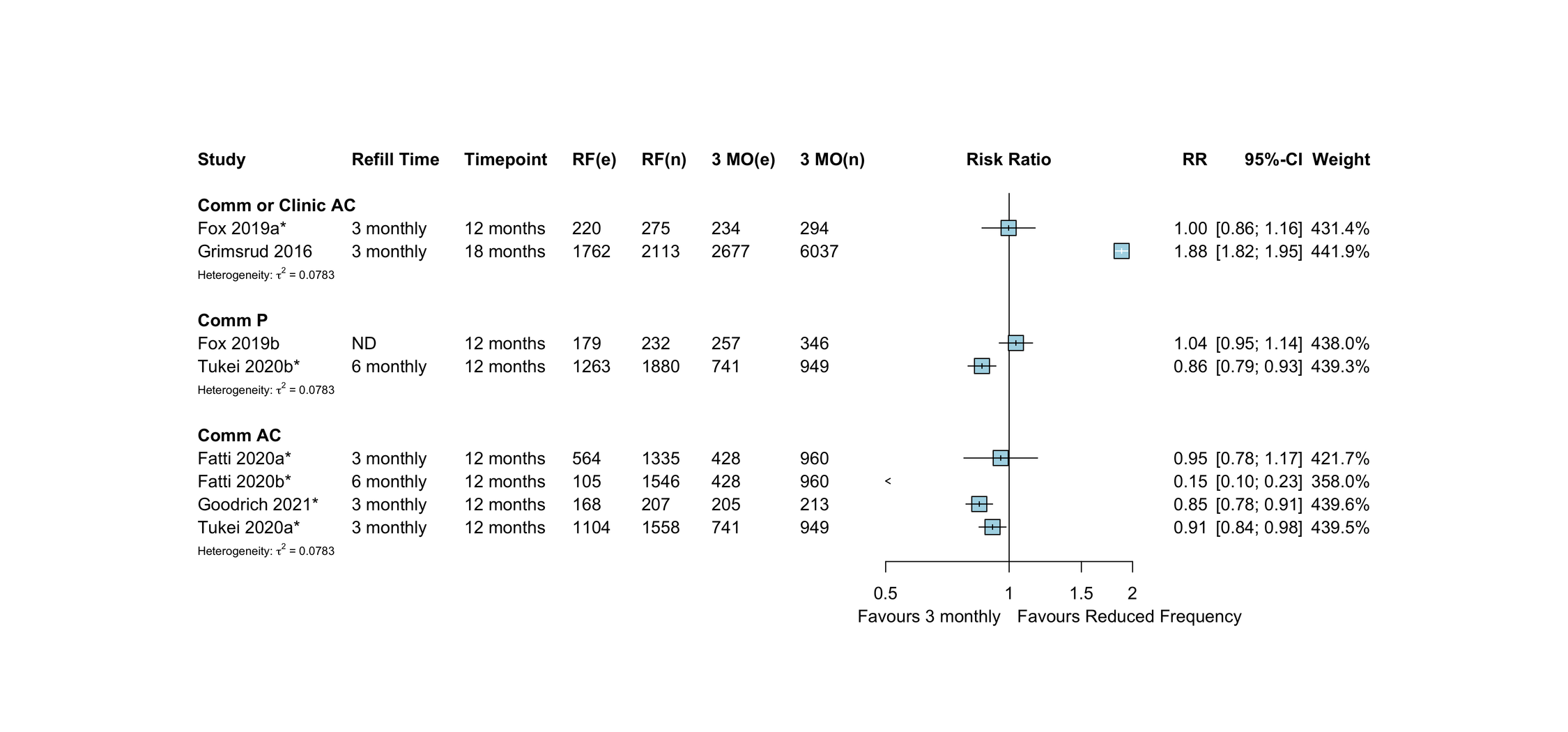

Supplement: S6 Fig — Fox 2019a and Fox 2019b were separated into an RCT and cohort design, respectively, based on the analysis described by the authors, where randomization was not preserved in the intervention arm in Fox 2019b. *Cluster-adjusted RR. 3 MO, 3-monthly; AC, adherence club; Comm, community; e, number of events; n, number of participants; RF, reduced frequency. (TIF) [file pmed.1003959.s008.tif]

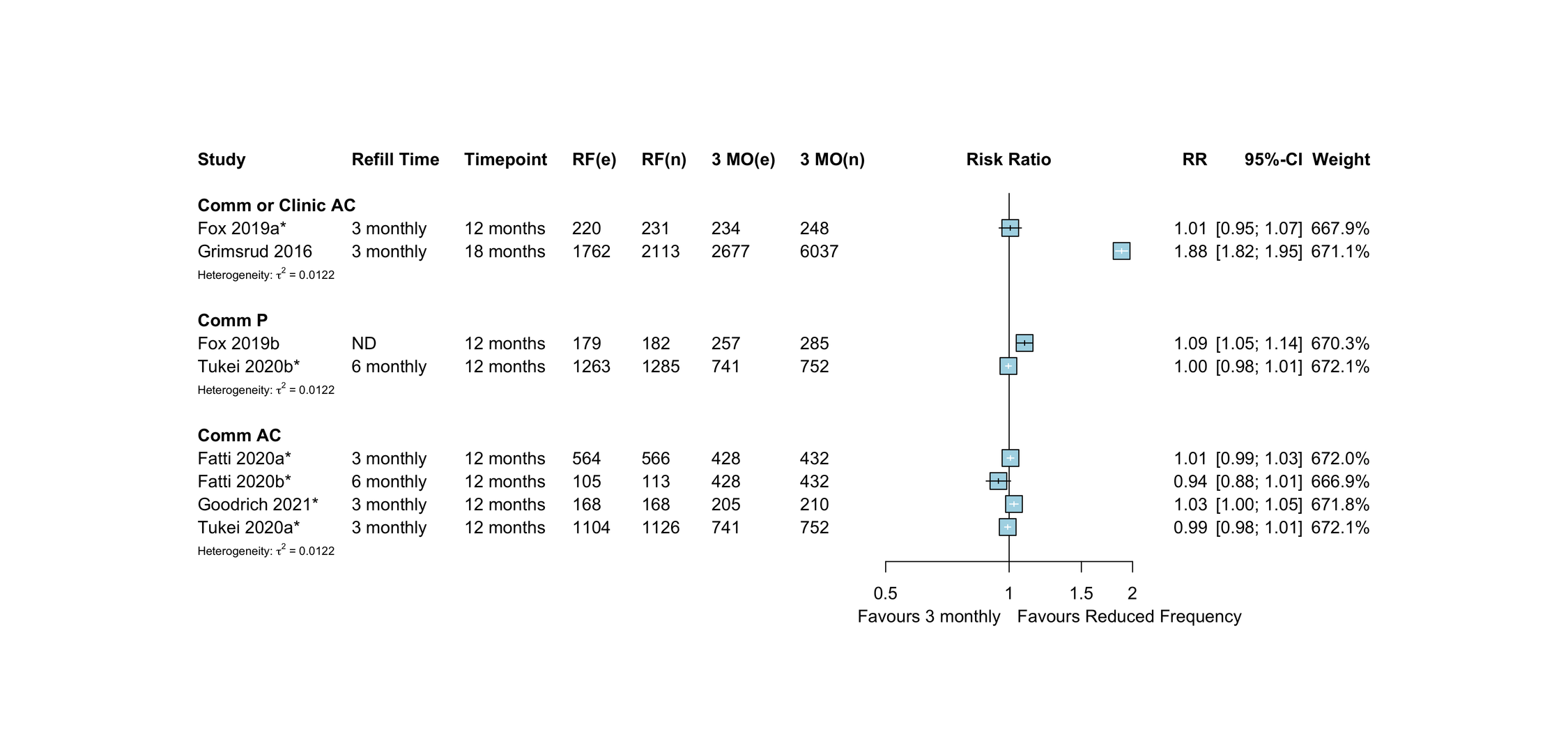

Supplement: S7 Fig — Fox 2019a and Fox 2019b were separated into an RCT and cohort design, respectively, based on the analysis described by the authors, where randomization was not preserved in the intervention arm in Fox 2019b. *Cluster-adjusted RR. 3 MO, 3-monthly; AC, adherence club; Comm, community; e, number of events; n, number of participants; RF, reduced frequency. (TIF) [file pmed.1003959.s009.tif]
