# Supplementary material for: Evaluation of HIV treatment outcomes with reduced frequency of clinical encounters and antiretroviral treatment refills: A systematic review and meta-analysis
Source: PLoS Med. 2022 Mar 22;19(3):e1003959. doi: 10.1371/journal.pmed.1003959 (PMC8982898; doi:10.1371/journal.pmed.1003959)
Supplement: S1 PRISMA Checklist — (DOCX) [file pmed.1003959.s010.docx]

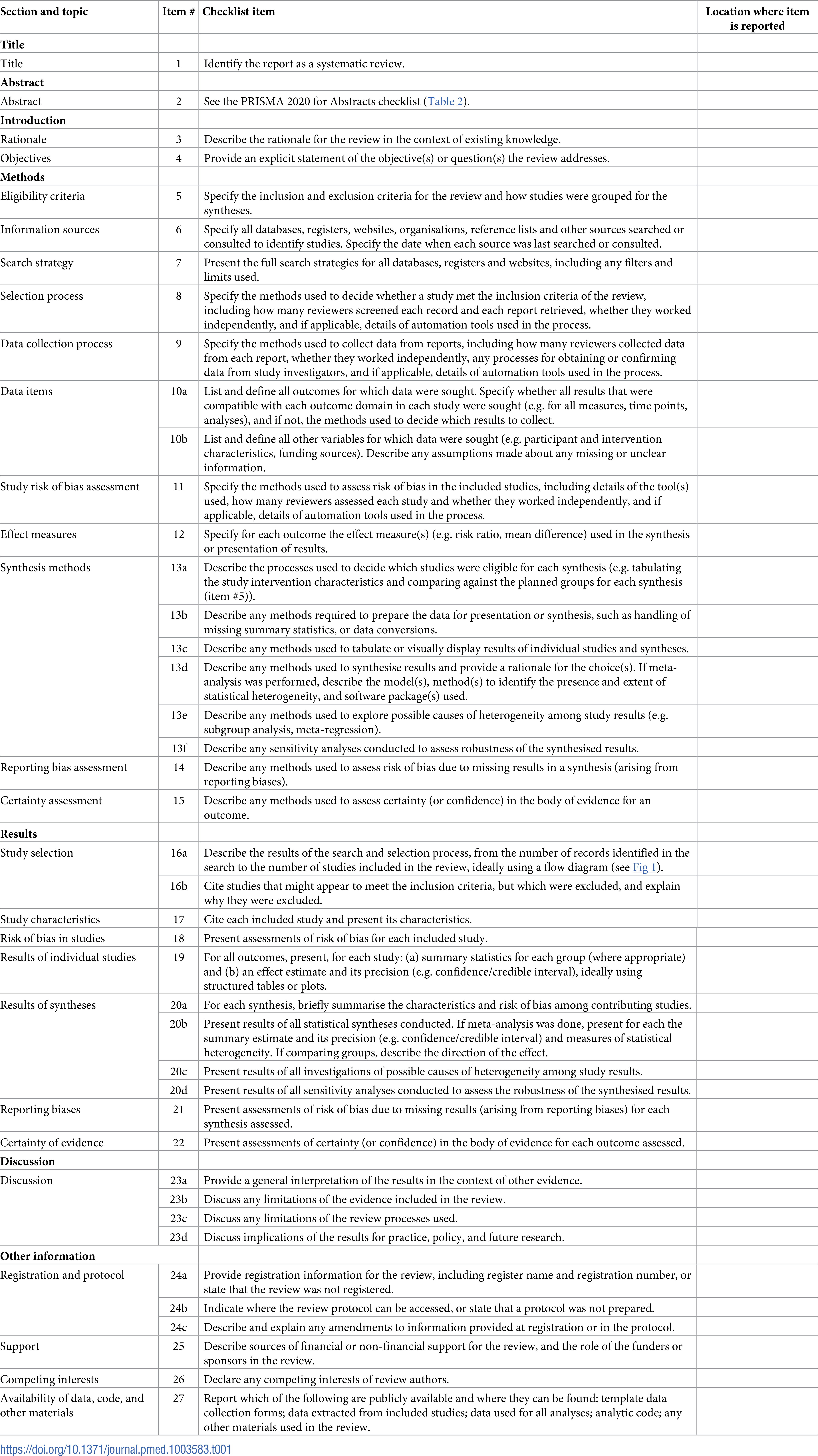


N/A

Results- Certainty of Evidence (GRADE) (P14-P15), Table 6, Table 7

Methods- Statistical analysis (P6)

Methods- Prior to P1

Methods- Prior to P1

Methods- Statistical analysis (P6)

Competing Interests

Financial Disclosure

Results- Table 2 includes all publicly available data used

Methods- Statistical analysis (P6)

Supporting Information – S1 Fig–S7 Fig

Supporting Information – S1 Fig–S7 Fig

Methods- Data extraction and quality assessment (P3)

N/A

Results- Fig 2-Fig 10; Supporting Information- S2 Table

Supporting Information - Deviations from protocol listed in PROSPERO

Methods- Eligibility criteria (P1), Outcome definition (P5); Supporting Information- S1 Table

Discussion- (P3-P5); Conclusion (P7)

Discussion- Limitations and Strengths (P6)

Discussion- Limitations and Strengths (P6)

Discussion- (P1-P2)

Results- Reduced clinical consultation frequency (P5-P10), Reduced ART refill dispensing frequency (P11-P13)

Results- Search and screening results (P1), Fig 1

Results- Search and screening results (P1), Fig 1

Results- Included studies (P2), Risk of Bias (P3)

Results- Included studies (P2), Table 2, Table 3

Methods- Intervention categorization (P4), Statistical analysis (P6)

Methods- Statistical analysis (P6)

Methods- Data extraction and quality assessment (P3), Statistical analysis (P6)

Methods- Statistical analysis (P6)

Methods- Eligibility criteria (P1)

Introduction (P3)

Introduction (P1, P2)

Results- Table 4

Methods- Intervention categorization (P4)

Methods- Data extraction and quality assessment (P3)

Methods- Data extraction and quality assessment (P3)

Methods- Data extraction and quality assessment (P3)

Supporting Information - Search Terms

Methods- Search strategy and selection criteria (P2)

Title page

Abstract
