# Supplementary material for: Evaluation of HIV treatment outcomes with reduced frequency of clinical encounters and antiretroviral treatment refills: A systematic review and meta-analysis
Source: PLoS Med. 2022 Mar 22;19(3):e1003959. doi: 10.1371/journal.pmed.1003959 (PMC8982898; doi:10.1371/journal.pmed.1003959)
Supplement: S1 Table — (DOCX) [file pmed.1003959.s011.docx]

**S1 Table. Outcome definitions by study**

| Study | Retention in Care | Viral suppression |
| --- | --- | --- |
| **Cassidy 2020** | Retained in care (any ART collection  (AC or clinic visit) at 24 months or within three months thereafter) | < 400 copies/mL |
| **Fatti 2020** | Retained in ART care (not lost to follow-up or deceased)  LTFU was defined in all arms as no ART collection for 90 days after the last missed scheduled ART collection date. | < 1000 copies/mL |
| **Fox 2019 a** | Retained in care (not lost to follow-up, transferred, or deceased) | < 400 copies/mL |
| **Fox 2019 b** | Retained in care (not lost to follow-up, transferred, or deceased) | < 400 copies/mL |
| **Goodrich 2021** | Completed study (not lost to follow-up, withdrawn, or deceased) | < 1000 copies/mL |
| **Grimsrud 2016** | N/A | < 1000 copies/mL |
| **Hoffman 2021** | Retention in care (﻿less than 60 consecutive days without ART at any point during follow-up) | N/A |
| **Nichols 2021** | Retained in care (Having a facility visit  between 9 and 15 months after model entry) | N/A |
| **Pasipamire 2018** | Retained in ART care (not lost to follow-up or deceased)  LTFU was defined as patients  without recorded visit for 120 days or more before database  closure. | N/A |
| **Tukei 2020a** | Retained in ART care (not lost to follow-up or deceased)  LTFU defined as missed a pick-up date for ART medication for more than 90 days after the last missed  Appointment. | < 1000 copies/mL |
| **Woodd 2014** | Present at end of trial (not lost to follow-up or deceased) | N/A |

N/A= Not applicable
