# Supplementary material for: Evaluation of HIV treatment outcomes with reduced frequency of clinical encounters and antiretroviral treatment refills: A systematic review and meta-analysis
Source: PLoS Med. 2022 Mar 22;19(3):e1003959. doi: 10.1371/journal.pmed.1003959 (PMC8982898; doi:10.1371/journal.pmed.1003959)
Supplement: S2 Table — (DOCX) [file pmed.1003959.s012.docx]

**S2 Table. Ascertainment of viral suppression by study**

|  | Reduced Frequency | | | | | | Three monthly facility based | | | | | |
| --- | --- | --- | --- | --- | --- | --- | --- | --- | --- | --- | --- | --- |
| Study | **Total Participants** | **VL Measured** | **VL Completion Rate** | **VL Suppressed** | **VL Suppressed Among All** | **VL Suppressed Among Those with VL Measured** | **Total Participants** | **VL Measured** | **VL Completion Rate** | **VL Suppressed** | **VL Suppressed Among All** | **VL Suppressed Among Those with VL Measured** |
| Fatti 2020 a | 1335 | 566 | 42% | 564 | 42% | 100% | 960 | 432 | 45% | 428 | 45% | 99% |
| Fatti 2020 b | 1546 | 113 | 7% | 105 | 7% | 93% | 960 | 432 | 45% | 428 | 45% | 99% |
| Fox 2019 a | 275 | 231 | 84% | 220 | 80% | 95% | 294 | 248 | 84% | 234 | 80% | 94% |
| Fox 2019 b | 232 | 182 | 78% | 179 | 77% | 98% | 346 | 285 | 82% | 257 | 74% | 90% |
| Goodrich 2021 | 207 | 168 | 90% | 168 | 90% | 100% | 213 | 210 | 99% | 205 | 96% | 98% |
| Grimsrud 2016 | 2113 | 2113 | - | 1762 | 83% | - | 6037 | 6037 | - | 2677 | 44% | - |
| Tukei 2020 a | 1558 | 1126 | 72% | 1104 | 71% | 98% | 949 | 752 | 79% | 741 | 78% | 99% |
| Tukei 2020 b | 1880 | 1285 | 68% | 1263 | 67% | 98% | 949 | 752 | 79% | 741 | 78% | 99% |
|  | **Reduced Frequency** | | | | | | **Six monthly facility based** | | | | | |
| Cassidy 2020 | 977 | 887 | 91% | 853 | 87% | 96% | 1173 | 999 | 85% | 969 | 83% | 97% |
