# Supplementary material for: Evaluation of HIV treatment outcomes with reduced frequency of clinical encounters and antiretroviral treatment refills: A systematic review and meta-analysis
Source: PLoS Med. 2022 Mar 22;19(3):e1003959. doi: 10.1371/journal.pmed.1003959 (PMC8982898; doi:10.1371/journal.pmed.1003959)
Supplement: S3 Table — (DOCX) [file pmed.1003959.s013.docx]

**S3 Table. Risk of Bias**

Retention: Randomized Controlled Trials

| **Study** | **Sequence Generation** | **Allocation Concealment** | **Blinding: Participants & Personnel** | **Blinding: Outcome Assessment** | **Attrition Bias** | **Selective Reporting** | **Other Bias** | **Cluster: Recruitment Bias** | **Cluster: Baseline Imbalance** | **Cluster: Loss of clusters** | **Cluster: Analysis** | **Overall ROB** |
| --- | --- | --- | --- | --- | --- | --- | --- | --- | --- | --- | --- | --- |
| **Cassidy 2020** | A computer-generated randomization list was generated  using the Randomize package in Stata, ensuring balance  between each arm, by a team member not involved in recruitment or enrollment. | Study staff drew an envelope containing the randomization outcome at the post consent AC meeting. | Participants and personnel were unblinded - blinding unlikely to bias the outcome in favor of the intervention. | Retention data were collected from routine AC registers completed  by facilitators. | LTFU was the analysis | Outcome outlined in protocol. | Some participants without documentation of recent viral suppression were included. AC patients who  missed a scheduled visit by more than five days were not always referred back to SOC and continued their care within their AC, with a higher proportion of these patients in SoC ACs.  During the first  year of the study a once-off non-routine four-month ART  refill was provided to all SoC AC patients. | Clusters were recruited as pre-existing ACs. When ≥90% of AC members present voted to participate in the study, the AC was eligible for inclusion. Intervention was delivered after cluster randomization. | Randomized ACs to ensure balance between community and facility ACs in each arm. No documented differences at baseline. | After  randomization, two ACs withdrew from the SoC: one did not  have enough members present to vote for continued participation, and in  the other <90% of members present voted for continued participation. | Risk differences were calculated using binomial generalized  estimating equations, using robust standard errors and specifying  clustering by AC. | Some concerns |
| **Fatti 2020a** | A computer-generated randomization list was generated restricted randomization for clusters using three strata: urban facilities, rural hospitals, and rural clinics | The method of concealment was not described. | Participants and personnel were unblinded - blinding unlikely to bias the outcome in favor of the intervention. | Measurement of outcome based on clinical records. | LTFU was the analysis | Outcome pre-specified in registration. |  | Intervention was delivered after cluster randomization with clusters stratified by facility type. | Baseline characteristics were well balanced between groups | No clusters were lost to follow up | Analysis used binomial population-averaged GEEs, with clustering by facility and robust standard errors. Model used small cluster size variance correction and randomization strata as a fixed-effect parameter. | Low risk |
| **Fatti 2020b** | A computer-generated randomization list was generated restricted randomization for clusters using three strata: urban facilities, rural hospitals, and rural clinics | The method of concealment was not described. | Participants and personnel were unblinded - blinding unlikely to bias the outcome in favor of the intervention. | Measurement of outcome based on clinical records. | LTFU was the analysis | Outcome pre-specified in registration. |  | Intervention was delivered after cluster randomization with clusters stratified by facility type. | Baseline characteristics were well balanced between groups | No clusters were lost to follow up | Analysis used binomial population-averaged GEEs, with clustering by facility and robust standard errors. Model used small cluster size variance correction and randomization strata as a fixed-effect parameter. | Low risk |
| **Fox 2019a** | Matched clinic pairs were randomized 1:1 by computer. | The method of concealment was not described. | Not all those eligible for the intervention necessarily received it, and if those at intervention sites were a select group of patients, this could create bias | Measurement of outcome based on clinical records. | LTFU was the analysis | Outcome pre-specified in registration | . | Intervention was delivered after cluster randomization. | Arms well balanced at baseline, with some small imbalances in CD4 count at ART initiation. Those in the intervention arm had been on treatment for substantially longer than the control arm. | No clusters were lost to follow up | Analysis accounted for clustering using a linear regression generalized estimating equation model. The model was then adjusted for differences in baseline covariates. | Some concerns |
| **Goodrich 2021** | The sequence generation method was not described. | The method of concealment was not described. | Participants and personnel were unblinded - blinding unlikely to bias the outcome in favor of the intervention | Measurement of outcome based on clinical records.  electronic point-of-care data collection forms and AC logbook | LTFU was the analysis | No registration. | Limited information regarding methods | Intervention was delivered after cluster randomization. | Study arms were well balanced at baseline and sublocations included in each study arm were matched based on their distance to the clinic. | No clusters were lost to follow up | Not described, but cluster adjusted in this analysis | Some concerns |
| **Hoffman 2021** | Randomisation was done by the study coordinator using a simple random allocation sequence generated by the study epidemiologist. | The method of concealment was not described. | Participants and personnel were unblinded - blinding unlikely to bias the outcome in favor of the intervention | Measurement of outcome based on clinical records. | LTFU was the analysis | Outcome outlined in protocol and registration. |  | Intervention was delivered after cluster randomization. | Clusters were matched on the basis of characteristics of interest, including country, ART cohort size, facility type, and region or province. | No clusters were lost to follow up | Analysis used facility-level clustering using GEEs to account for the study design and estimated robust standard errors. | Low risk |
| **Tukei 2020a** | The sequence generation method was not described. | The method of concealment was not described. | Participants and personnel were unblinded - blinding unlikely to bias the outcome in favor of the intervention | Measurement of outcome based on patient  files and monitoring tool used by study nurses. | LTFU was the analysis | Outcome outlined in protocol and registration. |  | Intervention was delivered after cluster randomization. | Baseline imbalance were present with respect to age and district.  . | No clusters were lost to follow up | Analysis used GEEs, specifying for clustering  by facility and using robust standard errors. | Low risk |
| **Tukei 2020b** | The sequence generation method was not described. | The method of concealment was not described. | Participants and personnel were unblinded - blinding unlikely to bias the outcome in favor of the intervention | Measurement of outcome based on patient  files and monitoring tool used by study nurses. | LTFU was the analysis | Outcome outlined in protocol and registration. |  | Intervention was delivered after cluster randomization. | Baseline imbalance were present with respect to age and district.  . | No clusters were lost to follow up | Analysis used GEEs, specifying for clustering  by facility and using robust standard errors. | Low risk |
| **Woodd 2014** | Clusters were defined in each stratum with a similar estimated number of HIV-infected  patients registered at the clinic, and then randomized 1:1 by drawing cards  from a concealed box. | Cards were drawn from concealed box to allocate clinic assignment. | Participants and personnel were unblinded - blinding unlikely to bias the outcome in favor of the intervention | Outcome assessors were not blinded but outcome measurement unlikely to be affected by detection bias. | LTFU was the analysis | Outcome was not reported as a specific outcome in the study but not affected by selective reporting |  | Intervention was delivered after cluster randomization | Baseline characteristics were well balanced between groups, however baseline CD4. cell counts were lower  in the home-based care arm | Not described | Not described, but cluster adjusted in this analysis. | Low risk |

Retention: Cohort Studies

| **Study** | **Representativeness of the exposed cohort** | **Selection of the non-exposed cohort** | **Ascertainment of exposure** | **Demonstration that outcome of interest was not present at start of study** | **Comparability of cohorts on the basis of the design or analysis controlled for confounders** | **Assessment of outcome** | **Follow-up long enough for outcome to occur** | **Adequacy of follow-up of cohorts** | **Overall ROB** |
| --- | --- | --- | --- | --- | --- | --- | --- | --- | --- |
| **Fox 2019b** | Enrolled those stable patients eligible and presenting at clinic | Sourced from similar clinics and sites were matched on district, total on ART, proportion virally suppressed, setting and location. | Ascertainment of exposure was based on  clinic register or patient files | Retention in care only relevant at follow-up | Adjusted for sex, age, and CD4 count at ART initiation in analysis | Assessment of outcome based on clinic records and a national electronic patient database. | Yes - follow-up time was likely sufficient | Retention in care described for both arms | High Quality |
| **Grimsrud 2016** | All stable patients were 16+ were able to join adherence club | Non-exposed from same ART clinic, but did not have to be stable | Ascertainment of exposure from  clinical database, the AC database, and the national lab database. | Retention in care only relevant at follow-up | Adjusted for age, sex, year of ART initiation, time-updated CD4, and viral load | Assessment of outcome based on clinic and study records | Yes - follow-up time was sufficient | Retention in care described for both arms | Fair Quality |
| **Nichols 2021a** | All enrolled were stable patients at sites with sufficient medical record data | Non-exposed sites were selected across similar sites in provinces that had not implemented DSD models | Ascertainment of exposure based on medical records from census of public sector ART facilities | Retention in care only relevant at follow-up | Matched stable patients on the basis of sex, age, urban/rural location, year of ART initiation | Assessment of outcome based on clinic records | Yes - follow-up time was likely sufficient | Retention in care described for both arms | High Quality |
| **Nichols 2021b** | All enrolled were stable patients at sites with sufficient medical record data | Non-exposed sites were selected across similar sites in provinces that had not implemented DSD models | Ascertainment of exposure based on medical records from census of public sector ART facilities | Retention in care only relevant at follow-up | Matched stable patients on the basis of sex, age, urban/rural location, year of ART initiation | Assessment of outcome based on clinic records | Yes - follow-up time was likely sufficient | Retention in care described for both arms | High Quality |
| **Nichols 2021c** | All enrolled were stable patients at sites with sufficient medical record data | Non-exposed sites were selected across similar sites in provinces that had not implemented DSD models | Ascertainment of exposure based on medical records from census of public sector ART facilities | Retention in care only relevant at follow-up | Matched stable patients on the basis of sex, age, urban/rural location, year of ART initiation | Assessment of outcome based on clinic records | Yes - follow-up time was likely sufficient | Retention in care described for both arms | High Quality |
| **Pasipamire 2018** | Eligibility was generally representative with of stable patients, but there was concern that health workers did not adhere to eligibility criteria and may have pressured patients to be included in one of the care models. | Participants in all three arms came from routine public health settings in Shiselweni region. However, eligibility differed by study arm. | Ascertainment of exposure was not described. | Retention in care only relevant at follow-up | Outcomes of all-cause attrition were adjusted for | Outcome ascertainment was not described. | Yes - follow-up time was likely sufficient | Retention in care described for both arms | Poor quality |

Viral Suppression: Randomized Controlled Trials

| **Study** | **Sequence Generation** | **Allocation Concealment** | **Blinding: Participants & Personnel** | **Blinding: Outcome Assessment** | **Attrition Bias** | **Selective Reporting** | **Other Bias** | **Cluster: Recruitment Bias** | **Cluster: Baseline Imbalance** | **Cluster: Loss of clusters** | **Cluster: Analysis** | **Overall ROB** |
| --- | --- | --- | --- | --- | --- | --- | --- | --- | --- | --- | --- | --- |
| **Cassidy 2020** | A computer-generated randomization list was generated  using the Randomize package in Stata, ensuring balance  between each arm, by a team member not involved in recruitment or enrollment. | Study staff drew an envelope containing the randomization outcome at the post consent AC meeting. | Participants and personnel were unblinded - blinding unlikely to bias the outcome in favor of the intervention. | Measurement of outcome based on laboratory data. | Attrition low in both arms- 6.4% in 6 monthly comparison arm and 7.4% in 12 monthly arm. | Outcome outlined in protocol. | Some participants without documentation of recent viral suppression were included. AC patients who  missed a scheduled visit by more than five days were not always referred back to mainstream care and continued their care within their AC, with a higher proportion of these patients in SoC ACs.  During the first  year of the study a once-off non-routine four-month ART  refill was provided to all SoC AC patients. | Clusters were recruited as pre-existing ACs. When ≥90% of AC members present voted to participate in the study, the AC was eligible for inclusion. Intervention was delivered after cluster randomization. | Randomized ACs to ensure balance between community and facility ACs in each arm. No documented differences at baseline. | After  randomization, two ACs withdrew from the SoC: one did not  have enough members present to vote for continued participation, and in  the other <90% of members present voted for continued participation. | Risk differences were calculated using binomial generalized  estimating equations, using robust standard errors and specifying  clustering by AC. | Some concerns |
| **Fatti 2020a** | A computer-generated randomization list was generated restricted randomization for clusters using three strata: urban facilities, rural hospitals, and rural clinics | The method of concealment was not described. | Participants and personnel were unblinded - blinding unlikely to bias the outcome in favor of the intervention. | Measurement of outcome based on clinical records. | Attrition low in both arms- 6.77% in comparison arm and 5.17% in 3 monthly arm. | Outcome pre-specified in registration. |  | Intervention was delivered after cluster randomization with clusters stratified by facility type. | Baseline characteristics were well balanced between groups | No clusters were lost to follow up | ITT analysis was conducted including all enrolled participants as allocated using a three-level outcome variable in a GEE framework and a multinomial logit model specified for clustering by facility. | Low risk |
| **Fatti 2020b** | A computer-generated randomization list was generated restricted randomization for clusters using three strata: urban facilities, rural hospitals, and rural clinics | The method of concealment was not described. | Participants and personnel were unblinded - blinding unlikely to bias the outcome in favor of the intervention. | Measurement of outcome based on clinical records. | Attrition low in both arms- 6.77% in comparison arm and 4.08% in 6 monthly arm. Differential viral outcome ascertainment of >10% | Outcome pre-specified in registration. | VL completion was high in Chitungwiza (96%) but substantially lower in all other districts (range: 14%–32%), particularly in rural areas. As no Chitungwiza facilities were allocated to 6MC, VL completion was lower in this arm. | Intervention was delivered after cluster randomization with clusters stratified by facility type. | Baseline characteristics were well balanced between groups | No clusters were lost to follow up | ITT analysis was conducted including all enrolled participants as allocated using a three-level outcome variable in a GEE framework and a multinomial logit model specified for clustering by facility. | High risk |
| **Fox 2019a** | Matched clinic pairs were randomized 1:1 by computer. | The method of concealment was not described. | Not all those eligible for the intervention necessarily received it, and if those at intervention sites were a select group of patients, this could create bias. | Measurement of outcome based on clinical records. | Attrition was 10.5% in intervention arm and 19.4% in the comparison arm | Outcome pre-specified in registration | . | Intervention was delivered after cluster randomization. | Arms were well balanced with respect to baseline demographics, but we saw some small imbalance in CD4 count at ART initiation and those in the intervention arm had been on treatment for substantially longer than the control arm. | No clusters were lost to follow up | Analysis accounted for clustering using a linear regression GEE model. The model was then adjusted for differences in baseline covariates. | Some concerns |
| **Goodrich 2021** | The sequence generation method was not described. | The method of concealment was not described. | Participants and personnel were unblinded - blinding unlikely to bias the outcome in favor of the intervention | Measurement of outcome based on clinical records.  electronic point-of-care data collection forms and AC logbook | Attrition was 1.4% in intervention arm and 18.8% in the comparison arm | No registration. | Limited information regarding methods | Intervention was delivered after cluster randomization. | Study arms were well balanced at baseline and sublocations included in each study arm were matched based on their distance to the clinic. | No clusters were lost to follow up | Not described, but cluster adjusted in this analysis | High risk |
| **Tukei 2020a** | The sequence generation method was not described. | The method of concealment was not described. | Participants and personnel were unblinded - blinding unlikely to bias the outcome in favor of the intervention | Measurement of outcome based on patient  files and monitoring tool used by study nurses. | Attrition low in both arms- 79.2% completed VL testing in comparison arm and 72.0% in 3 monthly refill intervention arm. | Outcome outlined in protocol and registration. |  | Intervention was delivered after cluster randomization. | Baseline imbalance were present with respect to age and district.  . | No clusters were lost to follow up | Analysis used GEEs, specifying for clustering  by facility and using robust standard errors. | Low risk |
| **Tukei 2020b** | The sequence generation method was not described. | The method of concealment was not described. | Participants and personnel were unblinded - blinding unlikely to bias the outcome in favor of the intervention | Measurement of outcome based on patient  files and monitoring tool used by study nurses. | Attrition low in both arms- 79.2% completed VL testing in comparison arm and 68.0% in 6 monthly refill intervention arm. Differential viral outcome ascertainment of >10% | Outcome outlined in protocol and registration. |  | Intervention was delivered after cluster randomization. | Baseline imbalance were present with respect to age and district.  . | No clusters were lost to follow up | Analysis used GEEs, specifying for clustering  by facility and using robust standard errors. | High risk |

Viral Suppression: Cohort studies

| **Study** | **Representativeness of the exposed cohort** | **Selection of the non-exposed cohort** | **Ascertainment of exposure** | **Demonstration that outcome of interest was not present at start of study** | **Comparability of cohorts on the basis of the design or analysis controlled for confounders** | **Assessment of outcome** | **Follow-up long enough for outcome to occur** | **Adequacy of follow-up of cohorts** | **Overall ROB** |
| --- | --- | --- | --- | --- | --- | --- | --- | --- | --- |
| **Fox 2019 b** | Enrolled those stable patients eligible and presenting at clinic | Sourced from similar clinics and sites were matched on district, total on ART, proportion virally suppressed, setting and location. | Ascertainment of exposure was based on  clinic register or patient files | Viral suppression present at start of study. | Adjusted for sex, age, and CD4 count at ART initiation in analysis | Assessment of outcome based on clinic records and a national electronic patient database. | Yes - follow-up time was likely sufficient | LTFU was low and comparable across arms | High Quality |
| **Grimsrud 2016** | All stable patients were 16+ were able to join adherence club | Non-exposed from same ART clinic, but did not have to be stable | Ascertainment of exposure was based on  CHC clinical database, the CAC database, and the National Health Laboratory Service database. | Viral suppression likely present at start of study | Adjusted for age, sex, year of ART initiation, time-updated CD4, and viral load | Assessment of outcome based on clinic and study records | Yes - follow-up time was sufficient | LTFU reported as adjusted hazard ratio | Fair Quality |

Mortality: Randomized Controlled Trials

| **Study** | **Sequence Generation** | **Allocation Concealment** | **Blinding: Participants & Personnel** | **Blinding: Outcome Assessment** | **Attrition Bias** | **Selective Reporting** | **Other Bias** | **Cluster: Recruitment Bias** | **Cluster: Baseline Imbalance** | **Cluster: Loss of clusters** | **Cluster: Analysis** | **Overall ROB** |
| --- | --- | --- | --- | --- | --- | --- | --- | --- | --- | --- | --- | --- |
| **Cassidy 2020** | A computer-generated randomization list was generated  using the Randomize package in Stata, ensuring balance  between each arm, by a team member not involved in recruitment or enrollment. | Study staff drew an envelope containing the randomization outcome at the post consent AC meeting. | Participants and personnel were unblinded - blinding unlikely to bias the outcome in favor of the intervention. | Outcome assessment was not described. | Attrition low in both arms- 6.4% in 6 monthly comparison arm and 7.4% in 12 monthly arm. | Outcome outlined in protocol. | Some participants without documentation of recent viral suppression were included. AC patients who  missed a scheduled visit by more than five days were not always referred back to mainstream care and continued their care within their AC, with a higher proportion of these patients in SoC ACs.  During the first  year of the study a once-off non-routine four-month ART  refill was provided to all SoC AC patients. | Clusters were recruited as pre-existing ACs. When ≥90% of AC members present voted to participate in the study, the AC was eligible for inclusion. Intervention was delivered after cluster randomization. | Randomized ACs to ensure balance between community and facility ACs in each arm. No documented differences at baseline. | After  randomization, two ACs withdrew from the SoC: one did not  have enough members present to vote for continued participation, and in  the other <90% of members present voted for continued participation. | Risk differences were calculated using binomial GEEs, using robust standard errors and specifying  clustering by AC. | Some concerns |
| **Fatti 2020a** | A computer-generated randomization list was generated restricted randomization for clusters using three strata: urban facilities, rural hospitals, and rural clinics | The method of concealment was not described. | Participants and personnel were unblinded - blinding unlikely to bias the outcome in favor of the intervention. | Measurement of outcome based on clinical records. | Attrition low in both arms- 6.77% in comparison arm and 5.17% in 3 monthly arm. | Outcome pre-specified in registration. |  | Intervention was delivered after cluster randomization with clusters stratified by facility type. | Baseline characteristics were well balanced between groups | No clusters were lost to follow up | Not described, but cluster adjusted in this analysis. | Low risk |
| **Fatti 2020b** | A computer-generated randomization list was generated restricted randomization for clusters using three strata: urban facilities, rural hospitals, and rural clinics | The method of concealment was not described. | Participants and personnel were unblinded - blinding unlikely to bias the outcome in favor of the intervention. | Measurement of outcome based on clinical records. | Attrition low in both arms- 6.77% in comparison arm and 4.08% in 6 monthly arm. | Outcome pre-specified in registration. |  | Intervention was delivered after cluster randomization with clusters stratified by facility type. | Baseline characteristics were well balanced between groups | No clusters were lost to follow up | Not described, but cluster adjusted in this analysis. | Low risk |
| **Goodrich 2021** | The sequence generation method was not described. | The method of concealment was not described. | Participants and personnel were unblinded - blinding unlikely to bias the outcome in favor of the intervention | Measurement of outcome based on clinical records.  electronic point-of-care data collection forms and AC logbook | Attrition was 1.4% in intervention arm and 18.8% in the comparison arm | No registration. | Limited information regarding methods | Intervention was delivered after cluster randomization. | Study arms were well balanced at baseline and sublocations included in each study arm were matched based on their distance to the clinic. | No clusters were lost to follow up | Not described, but cluster adjusted in this analysis | High risk |
| **Hoffman 2021** | Randomisation was done by the study coordinator using a simple random allocation sequence generated by the study epidemiologist. | The method of concealment was not described. | Participants and personnel were unblinded - blinding unlikely to bias the outcome in favor of the intervention. | Measurement of outcome based on clinical records. | Attrition was 13.6% in the comparison arm and 9.5% in the intervention arm. | Outcome outlined as part of primary outcome of retention in protocol and registration. |  | Intervention was delivered after cluster randomization. | Clusters were matched on the basis of known characteristics that might be associated with our outcomes of interest, including country, ART cohort size, facility type, and region or province. | No clusters were lost to follow up | Analysis used a facility-level clustering using GEEs to account for the study design and estimated robust standard errors. | Low risk |
| **Tukei 2020a** | The sequence generation method was not described. | The method of concealment was not described. | Participants and personnel were unblinded - blinding unlikely to bias the outcome in favor of the intervention | Measurement of outcome based on patient  files and monitoring tool used by study nurses. | Attrition low in both arms- 2.7% in comparison arm and 2.6% in 3 monthly refill intervention arm. | Outcome outlined as part of primary outcome of retention in protocol. |  | Intervention was delivered after cluster randomization. | Baseline imbalance were present with respect to age and district.  . | No clusters were lost to follow up | Analysis GEEs, specifying for clustering  by facility and using robust standard errors. | Low risk |
| **Tukei 2020b** | The sequence generation method was not described. | The method of concealment was not described. | Participants and personnel were unblinded - blinding unlikely to bias the outcome in favor of the intervention | Measurement of outcome based on patient  files and monitoring tool used by study nurses. | Attrition low in both arms- 2.7% in comparison arm and 4.3% in 6 monthly refill intervention arm. | Outcome outlined as part of primary outcome of retention in protocol.. |  | Intervention was delivered after cluster randomization. | Baseline imbalance were present with respect to age and district.  . | No clusters were lost to follow up | Analysis used GEEs, specifying for clustering  by facility and using robust standard errors. | Low risk |
| **Woodd 2014** | Clusters were defined in each  stratum with a similar estimated number of PLWH registered at the clinic, and randomized 1:1 by drawing cards  from a concealed box. | Cards were drawn from concealed box to allocate clinic assignment. | Participants and personnel were unblinded - blinding unlikely to bias the outcome in favor of the intervention. | Outcome assessors were not blinded but outcome measurement unlikely to be affected by detection bias. | Lost to follow-up was low- 2.3% in home-based arm and on arm and 2.4% in control arm. | Outcome was not reported as a specific outcome in the study but not affected by selective reporting |  | Intervention was delivered after cluster randomization | Baseline characteristics were well balanced between groups , however baseline CD4. cell counts were lower  in the home-based care arm | Not described | Not described | Low risk |

Mortality: Cohort studies

| **Study** | **Representativeness of the exposed cohort** | **Selection of the non-exposed cohort** | **Ascertainment of exposure** | **Demonstration that outcome of interest was not present at start of study** | **Comparability of cohorts on the basis of the design or analysis controlled for confounders** | **Assessment of outcome** | **Follow-up long enough for outcome to occur** | **Adequacy of follow-up of cohorts** | **Overall ROB** |
| --- | --- | --- | --- | --- | --- | --- | --- | --- | --- |
| **Pasipamire 2018** | Eligibility was generally representative with of stable patients, but there was concern that health workers did not adhere to eligibility criteria and may have pressured health workers to be included in one of the care models to benefit from less intensive clinic follow-up. | Participants in all three arms came from routine public health settings in Shiselweni region. However, eligibility differed by study arm. | Ascertainment of exposure was not described. | Participants were alive at the start of the study | No adjustment | Outcome ascertainment was not described. | Yes - follow-up time was likely sufficient | LTFU was low- 1.7% in CAG, 6.1% in comprehensive outreach, 4.2% in adherence clubs | Poor quality |

GEE=generalized estimating equation
